# Deep Learning-Based Assessment of European Seabass (*Dicentrarchus labrax*) Fillet Freshness and Quality Using Image Data

**DOI:** 10.3390/foods15193385

**Published:** 2026-09-23

**Authors:** Nermin Deliçay, Yesim Ozogul, Sakhi Mohammad Hamidy, Fethiye Takadaş, Yusuf Kuvvetli

**Affiliations:** 1 Department of Seafood Processing Technology, Cukurova University, 01330 Adana, Türkiye; nrmdelicay@gmail.com; 2 Department of Industrial Engineering, Istanbul Arel University, 34537 Istanbul, Türkiye; sakhihamidy@arel.edu.tr; 3 Department of Chemical Technology, Vocational School, University of Alanya, 07400 Antalya, Türkiye; fethiye.takadas@alanyauniversity.edu.tr; 4 Department of Industrial Engineering, Cukurova University, 01330 Adana, Türkiye; ykuvvetli@cu.edu.tr

**Keywords:** European seabass, freshness assessment, non-destructive quality assessment, deep learning

## Abstract

Artificial intelligence and image-based deep learning offer promising non-destructive approaches for rapid fish quality assessment. This study evaluated the potential of deep learning models to classify freshness-related sensory attributes and predict color parameters of European seabass (*Dicentrarchus labrax*) fillets using image data acquired under controlled illumination. Seven sensory quality parameters and six color attributes were assessed. A structured image dataset was obtained at a fixed camera-to-sample distance of 45 cm, and 65 independently trained models, comprising 35 classification and 30 regression models, were developed using DenseNet121, InceptionV3, MobileNet, VGG16, and VGG19 architectures. Model training and evaluation were performed using group-based 10-fold cross-validation to ensure that images from the same fillet were not shared between the training and test sets. Classification performance depended strongly on both architecture and target parameter. DenseNet121 achieved the highest accuracy for purchasability (92.60%), water release (90.20%), odor (75.60%), color (77.40%), and disintegration (87.70%), whereas MobileNet performed best for texture (81.20%) and total score (57.70%). Purchasability was the most predictable sensory parameter across architectures, with a mean accuracy of 86.84%, whereas total score was the least predictable at 45.64%. In the regression tasks, DenseNet121 achieved the lowest MAE for a* (0.680), L* (2.467), hue (0.961), and whiteness (2.357), while MobileNet performed best for b* (1.200) and chroma (1.024). Grad-CAM visualizations indicated that model activations were predominantly associated with the fillet region, although more difficult classes showed more fragmented and occasionally edge- or background-associated activation patterns. Overall, DenseNet121 provided the strongest and most consistent performance, ranking first for nine of the 13 prediction targets. These findings demonstrate the potential of RGB imaging and deep learning for rapid, objective, and non-destructive assessment of selected freshness and quality attributes of European seabass fillets.

## 1. Introduction

The growing global population, together with rapid urbanization and improving living standards, is steadily increasing the demand for animal-derived protein and placing greater pressure on food production systems. Aquatic foods have become an increasingly important component of global diets, with aquaculture playing a central role in meeting this rising demand. According to the FAO State of World Fisheries and Aquaculture 2026 report, global fisheries and aquaculture production reached a record 235 million tonnes in 2024, including 195 million tonnes of aquatic animals, while aquaculture production exceeded 100 million tonnes for the first time and now accounting for more than half of global aquatic animal production [1]. These trends highlight the critical role of sustainable aquaculture in strengthening global food security, nutrition, and resilient food systems.

Fish and fishery products are widely recognized as nutritious foods owing to their high-quality proteins, essential amino acids, vitamins, minerals, and long-chain omega-3 polyunsaturated fatty acids, particularly eicosapentaenoic acid (EPA) and docosahexaenoic acid (DHA) [2,3]. Increasing consumer awareness of the health benefits associated with fish consumption has contributed to a steady rise in global fish demand, making quality preservation and freshness evaluation increasingly important throughout the production and distribution chain [4]. In particular, European seabass (*Dicentrarchus labrax*) is one of the most valuable marine aquaculture species in the Mediterranean region because of its desirable sensory characteristics, high nutritional value, and strong commercial demand [5].

Despite its nutritional advantages, fish remains one of the most perishable food commodities. Fish deterioration is a complex process involving chemical, physical, and microbiological changes that progressively alter sensory and organoleptic properties, with the rate and extent of quality loss varying among species and being strongly influenced by both pre- and post-harvest factors, including environmental and rearing conditions, handling stress, slaughtering methods, processing practices, and storage conditions [6,7].

Freshness evaluation has traditionally relied on sensory analysis, microbiological examination, and physicochemical indicators, including total volatile basic nitrogen (TVB-N), trimethylamine (TMA), pH, thiobarbituric acid reactive substances (TBARS), K-value, color, and texture measurements [8,9,10]. Although these analytical techniques provide reliable information, they are generally destructive, labor-intensive, time-consuming, and unsuitable for rapid industrial inspection. Consequently, there is an increasing demand for non-destructive, automated, and real-time techniques capable of accurately assessing fish freshness throughout the seafood supply chain [11,12].

Recent developments in artificial intelligence (AI), computer vision, and deep learning have created new opportunities for food quality assessment. Compared with conventional machine learning approaches that depend on manually extracted image features, deep learning algorithms automatically learn hierarchical visual representations from raw images, enabling more robust and accurate classification of complex quality attributes [13,14,15]. Convolutional neural networks (CNNs) and their advanced variants have demonstrated remarkable performance in food inspection tasks, including defect detection, species identification, maturity estimation, and freshness evaluation [16,17]. These technologies offer rapid, objective, and non-destructive alternatives that can be integrated into intelligent quality control systems for the food industry.

Within the seafood sector, image-based deep learning has primarily focused on whole fish by exploiting external characteristics such as eye appearance, gill color, skin brightness, and skin mucus. However, fish is also widely marketed as fillets, in which these external anatomical indicators are absent. Consequently, freshness-related changes in fillets are reflected mainly through more subtle variations in surface color, moisture distribution, muscle texture, and structural appearance, making visual freshness assessment and automated classification more challenging. Building on our previous study, which demonstrated the feasibility of predicting fish freshness and purchasability from multi-angle images of whole fish using deep learning [15], the present study extends this approach to European seabass (*Dicentrarchus labrax*) fillets. Compared with whole fish, fillets lack external anatomical features such as the eyes and gills that are commonly used as visual indicators of freshness, while storage-induced changes in color, surface texture, and moisture distribution can be more subtle and difficult to detect. Therefore, this study aimed to develop and evaluate deep learning models for the freshness classification of European seabass fillets using RGB image data acquired under controlled imaging conditions.

## 2. Materials and Methods

### 2.1. Sample Preparation and Storage

The materials used in this study were specifically prepared for the experimental procedures. Ten European seabass (*Dicentrarchus labrax*) were purchased as fillets from a local market. From the day of purchase, the fillets were stored under refrigerated conditions until 4–5 days after they were considered spoiled. During this period, quality measurements and image acquisition procedures were performed daily. To minimize sample-to-sample variability, each fillet was individually numbered, and the same fillets were imaged daily in the same predetermined order throughout the storage period under standardized imaging conditions.

### 2.2. Sensory Quality Assessment

The sensory evaluation was conducted by a panel of ten trained panelists familiar with the sensory characteristics of the samples. The panel consisted of ten male and female volunteers aged 25–55 years. All panelists provided verbal consent to participate in the study. Prior to the evaluation, all participants underwent a training session to ensure consistency in scoring, during which they were familiarized with the evaluation criteria adapted from Bonilla et al. [18]. In addition to the parameters proposed by the researchers, two supplementary parameters, namely the total score and purchasability status, were included in the evaluation procedure. The total quality score represents the sum of the scores assigned to all individual quality parameters, whereas the purchasability parameter indicates whether the product is considered spoiled and unsuitable for consumption. According to the assessment system, products with a total score below 7 are considered suitable for consumption. Conversely, products with a total score of 7 or higher are regarded as spoiled and therefore unsuitable for consumption. The purchasability status of each sample was evaluated independently of its calculated total quality score. The parameters assessed during the raw sensory evaluation and their corresponding scoring criteria are presented in Table 1.

The total quality scores and purchasability assessments of the 10 fillet samples are comparatively presented in Figure 1. For each sample, the figure shows the total quality score obtained from the daily measurements together with the purchasability status assigned by the expert, indicating whether the product was considered suitable for purchasing (accept) or not (reject).

### 2.3. Color Analysis

For color analysis, the approach proposed by Hunter [19] was adopted. In this method, the L*, a*, and b* coordinates of the CIELab color space were measured, and the corresponding whiteness, chroma, and hue values were subsequently calculated. All color measurements were performed using a CR-400 device (Konica Minolta, Osaka, Japan).

The L* value represents lightness, ranging from 0 for black to 100 for white. Positive a* values indicate redness, whereas negative a* values indicate greenness, within a range of −60 to +60. Similarly, positive b* values represent yellowness, while negative b* values represent blueness, also within a range of −60 to +60. Based on the measured L*, a*, and b* values in the CIELab system, whiteness, chroma, and hue were calculated using Equations (1)–(3), as described by Hunter [19].

(1) $$Whiteness=100-{\left[{\left(100-L^{*}\right)}^{2}+a^{*2}+b^{*2}\right]}^{\frac{1}{2}}$$

(2) $$Chroma={\left[a^{*2}+b^{*2}\right]}^{\frac{1}{2}}$$

(3) $$Hue=arctan(b^{*}/a^{*})$$

For each fillet sample, color measurements were performed at three different points. Since the measured L*, a*, and b* values, together with the calculated whiteness, chroma, and hue values, serve as indicators of fish freshness, estimating the mean values of these parameters also constituted one of the objectives of this study.

### 2.4. Image Acquisition

Because image acquisition was performed at different times of the day, the imaging procedure was conducted in a specially designed room to prevent variations in natural daylight between different days and times from affecting image quality. To eliminate external light, the windows were covered with black opaque film, thereby isolating the experimental environment from daylight. A custom-built imaging stand was mounted on the table. The stand consisted of a rigid rectangular frame positioned perpendicular to the table surface. The camera was fixed to the upper section of the stand, allowing all samples to be photographed vertically. To prevent angular displacement over time, the stand was secured at multiple points. The distance between the camera lens and the sample surface was maintained at 45 cm throughout the image acquisition process.

Illumination was provided by LED strips installed along the inner surfaces of the stand. To ensure consistent lighting conditions, 5050- and 2835-type surface-mounted device (SMD) LEDs with a density of three LEDs per 5 cm and a color temperature of 3000 K were used. The LED strips were fixed along the frame so that they surrounded the imaging area and provided uniform illumination. A schematic representation of the custom-built imaging stand is presented in Figure 2.

Images were acquired using a Canon EOS 60D digital camera (Canon Inc., Tokyo, Japan) equipped with an APS-C CMOS sensor and an 18–200 mm lens kit. To maintain a consistent magnification ratio across all photographs, the zoom setting was fixed throughout image acquisition. Considering the background color and illumination intensity, the camera settings were fixed at an aperture of f/4.5, a shutter speed of 1/50 s, and an ISO sensitivity of 100. The acquired images had a resolution of 3456 × 2308 pixels.

For each fillet sample, quality assessment and image acquisition were performed daily over an 18-day period, predominantly during the morning hours. At the advanced stages of spoilage, additional images were also captured at noon and in the evening. During the first 5 days, one image was acquired per day, whereas from Day 6 onward, three images were recorded for each fillet to better document the progression of spoilage. However, on one public holiday, only two images were acquired, in the morning and evening. Consequently, a total of 43 images were obtained from each fillet, resulting in 430 images in the final dataset. The distribution of the 430 images among the classes corresponding to each sensory quality parameter is presented in Table 2.

Figure 3 presents representative images of the same fillet obtained on three different storage days, ranging from the fresh state to a visibly advanced stage of spoilage. This temporal sequence enables direct visual comparison of freshness loss and reveals the gradual changes in color, brightness, and surface characteristics.

In summary, the dataset used in this study comprised the acquired images and the results of the raw sensory and color analyses conducted by experts. The images included in the dataset were used to predict the analytical results in accordance with the primary objective of the study. To achieve this objective, deep learning approaches were employed as the principal methodological framework.

### 2.5. Deep Learning Model Development

In this study, artificial intelligence-based methods were employed to determine various quality attributes from images of seabass fillets. The primary focus was the prediction of purchasability status, which represents a critical criterion in the evaluation of fillet quality. The investigated quality indicators were divided into two main groups according to their data structure. The outcomes of the raw sensory evaluation were treated as discrete categorical variables, whereas the measurements obtained from color analysis were considered continuous numerical variables. The color-related parameters evaluated in this study consisted of continuous values. Because these parameters are sufficiently sensitive to reflect color changes occurring even during the early stages of spoilage, they may facilitate a more detailed and accurate representation of changes throughout the freshness deterioration process. Accordingly, regression analysis was adopted rather than categorizing these measurements, thereby preserving their original measurement resolution and enabling more precise predictions of continuous quality indicators. Based on this distinction, classification models were used to predict sensory quality parameters, including purchasability status, whereas regression models were employed to estimate the color parameters.

Five transfer learning architectures (DenseNet121, InceptionV3, MobileNet, VGG16, and VGG19) were evaluated for both the classification and regression tasks. These architectures were selected based on their demonstrated effectiveness in image analysis applications and their distinct feature-extraction capabilities. DenseNet121 promotes feature reuse through dense connections between layers, which may facilitate the detection of subtle details in fillet surface texture. MobileNet, owing to its computationally efficient architecture and optimized feature-extraction mechanism, is well suited to identifying color transitions and gradient based variations. Similarly, the VGG and Inception based models can process visual information at different depths and spatial scales, thereby generating robust hierarchical feature representations. These capabilities are particularly relevant for distinguishing visual indicators associated with fish quality, including changes in brightness, surface structure, and color. The complementary feature extraction characteristics of the selected architectures therefore make them suitable candidates for image-based prediction of seafood freshness and quality.

Dense Convolutional Networks (DenseNet), introduced by Huang et al. [20] are based on a connectivity pattern in which each layer is directly connected to all subsequent layers in the network. In the DenseNet121 architecture, which employs feature-map concatenation, each layer receives the feature maps generated by all preceding layers as input. This connectivity pattern enables previously learned features to be reused in later parts of the network, thereby improving parameter efficiency. Consequently, DenseNet121 can achieve high predictive performance with a relatively small number of parameters, while its enhanced gradient flow helps alleviate the vanishing-gradient problem. The principal advantages of this architecture, which has demonstrated strong performance particularly in object recognition and image-classification tasks, include feature reuse, parameter efficiency, and improved gradient propagation. However, the continuous concatenation of feature maps across layers may increase memory requirements, particularly in large-scale networks, and may therefore complicate computational resource management [20].

InceptionV3, developed by Szegedy et al. at Google, is based on Inception modules designed to optimize the depth and width of a network simultaneously. The model comprises approximately 42 layers and employs various factorization strategies to reduce computational cost. For example, large convolutional filters are decomposed into smaller filters. A 7 × 7 convolution can therefore be replaced by a sequence of smaller convolutions, while an *n* × *n* convolution can be factorized into consecutive 1 × *n* and *n* × 1 operations. This approach is intended to reduce the number of parameters and computational burden while preserving the representational capacity of the model. InceptionV3 also incorporates auxiliary classifiers that support learning at intermediate stages of the network and regularization techniques, such as label smoothing, to reduce the risk of overfitting. The architecture has achieved successful results in applications such as medical-image assessment and the processing of large-scale image datasets. Although its ability to attain high accuracy with fewer than 25 million parameters represents an important advantage, its complex and highly customized architectural design may make adaptation and implementation more challenging [21,22,23].

MobileNet, developed by Howard et al. [24] in 2017, is a deep-learning architecture designed primarily for use on mobile devices and embedded systems, with an emphasis on low latency and reduced computational cost. Its defining characteristic is the use of depth wise separable convolutions, which divide a standard convolution operation into two separate stages. In the first stage, depth wise convolution is performed by applying an individual filter to each input channel. The resulting outputs are then combined through a 1 × 1 pointwise convolution. This 28-layer architecture reduces computational cost by approximately eight- to nine-fold compared with standard convolution operations while limiting the associated loss in predictive accuracy. MobileNet has been applied to various image-processing tasks, including object detection, facial-feature recognition, and large-scale geolocation. In addition, two general hyperparameters, referred to as the width multiplier and resolution multiplier, allow the model size, processing speed, and accuracy to be adjusted according to the requirements of a particular application [24].

The VGG16 architecture was developed in 2014 by Simonyan and Zisserman [25], researchers at the Visual Geometry Group of the University of Oxford. In contrast to earlier architectures such as AlexNet, which employed large 11 × 11 or 7 × 7 filters, VGG16 uses sequentially arranged 3 × 3 convolutional filters. This design increases network depth while enabling the extraction of stronger and more detailed visual representations. VGG16 consists of 16 weighted layers, including 13 convolutional layers and three fully connected layers. Each convolutional block is followed by a 2 × 2 max-pooling operation. Owing to its regular, simple, and homogeneous architectural design, VGG16 has been successfully applied in a variety of fields, including image classification, skin-cancer diagnosis, and the detection of rice-leaf diseases. One of its most important contributions was demonstrating the significance of network depth in learning visual features. Nevertheless, its approximately 138 million parameters and computational cost of approximately 15.3 billion floating-point operations make its implementation challenging, particularly on mobile devices with limited memory and processing capacity [22,25,26].

VGG19, a deeper variant of VGG16, was also introduced by Simonyan and Zisserman [25] in 2014. The architecture contains 19 weighted layers, consisting of 16 convolutional layers and three fully connected layers. Similar to VGG16, VGG19 employs sequentially arranged 3 × 3 convolutional filters to increase network depth. For example, the receptive field of a single 7 × 7 filter can be achieved by applying three consecutive 3 × 3 filters. This strategy introduces a greater number of nonlinear transformations and enables the network to learn complex image patterns in greater detail. VGG19 has been widely used in applications such as image-based emotion analysis, fault diagnosis in rotating machinery, and the extraction of complex visual features. Although its deeper structure provides a high feature-extraction capacity, its approximately 144 million parameters and computational complexity of approximately 19.6 billion floating-point operations substantially increase training and implementation costs. Consequently, training and deploying VGG19 require greater computational resources than VGG16 [25,26,27].

The overall methodology of the study is illustrated in Figure 4. Images of seabass fillets were used as inputs to five different transfer learning architectures: DenseNet121, InceptionV3, MobileNet, VGG16, and VGG19. The target quality attributes were divided into two groups according to their data types and were modelled independently. Seven categorical parameters obtained from the raw sensory evaluation were predicted using classification models. Accordingly, 35 classification models were developed by combining five architectures with seven sensory parameters. In contrast, 6 continuous numerical parameters derived from the color analysis were estimated using regression models, resulting in 30 regression models based on five architectures and 6 color parameters. Thus, a total of 65 independent deep learning models were trained and evaluated to predict the sensory and instrumental quality characteristics of the fillet samples from their images. Among the color analysis parameters, Avg_L* denotes the mean L* value obtained from measurements taken at three points. The same naming convention was applied to the remaining color parameters.

### 2.6. Performance Evaluation Metrics

To evaluate the performance of the classification models, four metrics were used: accuracy, precision, recall, and F1-score. The mathematical expressions corresponding to these metrics are presented in Equations (4)–(7). In these equations, TP denotes true positives, TN denotes true negatives, FP denotes false positives, and FN denotes false negatives [28].

(4) $$Accuracy=\frac{TP+TN}{TP+TN+FP+FN}$$

(5) $$Precision=\frac{TP}{TP+FP}$$

(6) $$Recall=\frac{TP}{TP+FN}$$

(7) $$F1\text{-}Score=2\times \frac{Precision\times Recall}{Precision+Recall}$$

The L*, a*, and b* color parameters are highly sensitive to small variations. Therefore, the performance of the regression models developed to predict these parameters was assessed using tolerance-based error criteria. This approach evaluates prediction accuracy by assigning the absolute difference between the predicted and measured values to predefined tolerance categories. The tolerance intervals, adapted from research done by Şahin et al. [29], were defined as follows:

- Excellent: absolute error ≤ 0.1 for L*, a*, and b*.
- Good: absolute error > 0.1 and ≤0.5 for L*, and >0.1 and ≤0.3 for a* and b*.
- Medium: absolute error > 0.5 and ≤1.0 for L*, and >0.3 and ≤0.6 for a* and b*.
- Acceptable: predictions with absolute errors ≤ 1.0 for L* and ≤0.6 for a* and b* were considered acceptable overall.
- Rejected: predictions with absolute errors > 1.0 for L* or >0.6 for a* and b* were classified as unacceptable.

In addition to the tolerance-based evaluation, mean absolute error (MAE) was used to assess the performance of all regression models, including those developed for predicting L*, a*, and b*. MAE represents the average magnitude of the absolute differences between the predicted and measured values, irrespective of error direction. It therefore provides a direct and interpretable measure of prediction accuracy, with lower MAE values indicating better model performance. The mathematical expression for MAE is presented in Equation (8), where $y_{i}$ denotes the measured value, ${\hat{y}}_{i}$ represents the predicted value, and n is the total number of observations [30].

(8) $$MAE=\frac{1}{n}\sum_{i=1}^{n}\left|y_{i}-{\hat{y}}_{i}\right|$$

### 2.7. Model Training Configuration

A All computational analyses were performed using Python 3.14.7. Deep learning model development and training were conducted using TensorFlow 2.20.0-rc0 and Keras 3.11.0. Data processing was performed using NumPy 2.2.5 and Pandas 2.2.3, while model evaluation and cross-validation were performed using scikit-learn 1.6.1. Excel-based data handling was performed using OpenPyXL 3.1.5. Image processing and visualization were performed using OpenCV 4.11.0.86, Pillow 11.2.1, Matplotlib 3.10.1, and scikit-image 0.25.2, where applicable.

The architectural and training configurations used for the regression and classification tasks are summarized in Table 3. For all transfer-learning architectures, the original classification head was removed and replaced with a global average-pooling layer followed by a fully connected layer containing 64 neurons with ReLU activation. The final output layer was configured according to the prediction task: a single neuron with linear activation was used for regression, whereas a SoftMax layer with a task-dependent number of neurons was used for classification. Model training and evaluation were performed using a group-based 10-fold cross-validation procedure based on the 10 individual fish. In each fold, all images belonging to one fish were held out exclusively as the test set, while the remaining nine fish were used for model development. Of these nine fish, one was further separated as the validation set and the remaining eight were used for training. Thus, each fish served as the independent test group once during the 10-fold procedure, preventing images from the same fish from being shared between the training and test sets. Model training was performed for a maximum of 30 epochs with a batch size of 8, and early stopping was applied by monitoring the validation loss with a patience of five epochs and restoring the best-performing weights. To ensure a consistent comparison among the architectures, all models were trained using the same input dimensions, group-based data-partitioning strategy, optimizer, batch size, and training procedure. Model performance was calculated separately for each of the 10 independently held-out fillet folds. To quantify inter-fold variability, the primary classification metric (accuracy) and regression metric (MAE) were summarized as mean ± standard deviation (SD) across the 10 test folds.

## 3. Results and Discussion

### 3.1. Sensory Quality Parameters Prediction Performance

Table 4 presents the performance of the 35 classification models developed by combining five transfer-learning architectures with seven sensory quality parameters. The models were ranked separately for each target parameter according to classification accuracy. To account for variability among the independently held-out fillets, classification accuracy is reported as mean ± SD across the 10 cross-validation folds. In addition to accuracy, macro-averaged precision, recall, and F1-score values were reported to provide a more balanced assessment of model performance across classes.

Overall, DenseNet121 and MobileNet showed the strongest performance across the evaluated parameters. DenseNet121 achieved the highest accuracy for water release, odor, color, disintegration, and purchasability, with accuracies of 90.20%, 75.60%, 77.40%, 87.70%, and 92.60%, respectively. MobileNet was the best-performing architecture for texture and total-score classification, reaching accuracies of 81.20% and 57.70%, respectively. InceptionV3 did not achieve the highest accuracy for any of the evaluated parameters, although it remained competitive for several classification tasks.

Among all architecture–parameter combinations, the highest overall classification performance was obtained by DenseNet121 for purchasability, with an accuracy of 92.60%. For this parameter, the model also achieved a precision of 0.914, recall of 0.899, and F1-score of 0.891, indicating strong and relatively balanced performance across classes. DenseNet121 also performed strongly for disintegration, achieving an accuracy of 87.70% and an F1-score of 0.864. By contrast, total score was the most difficult parameter to classify. Even the best-performing model, MobileNet, achieved an accuracy of only 57.70% and an F1-score of 0.304 for this parameter.

VGG16 and VGG19 generally produced lower classification performance than DenseNet121 and MobileNet, particularly in terms of macro-averaged precision, recall, and F1-score. This difference was especially pronounced for total-score classification, where VGG16 achieved an accuracy of 38.40% and an F1-score of 0.124, while VGG19 achieved an accuracy of 30.20% and an F1-score of only 0.080. Nevertheless, VGG16 showed comparatively stronger performance for some parameters, particularly purchasability and water release, with accuracies of 85.10% and 83.50%, respectively. These findings further demonstrate that classification accuracy should be interpreted together with precision, recall, and F1-score when evaluating balanced class-level performance.

The parameter-specific accuracy values of the classification models are visualized as a heatmap in Figure 5. The heatmap clearly demonstrates that model performance varied according to both the selected architecture and the target quality parameter. Overall, the highest accuracy values were predominantly observed for DenseNet121 and MobileNet, whereas VGG16 and VGG19 generally exhibited lower performance across the evaluated parameters.

When averaged across the seven classification parameters, DenseNet121 achieved the highest mean accuracy of 79.30%, followed by MobileNet at 78.40% and InceptionV3 at 72.80%. The corresponding mean accuracies of VGG16 and VGG19 were lower, at 67.30% and 62.70%, respectively. Nevertheless, no single architecture consistently yielded the best result for every quality parameter. DenseNet121 achieved the highest accuracy for water release, odor, color, disintegration, and purchasability, whereas MobileNet produced the highest accuracy for texture and Total Score. These results indicate that the effectiveness of each architecture depended on the visual characteristics associated with the target quality parameter.

Figure 6 compares the overall predictability of the raw sensory quality parameters based on the mean accuracy obtained across all transfer-learning architectures. Purchasability was the most accurately predicted parameter, with a mean accuracy of 86.84%, followed by water release at 84.94% and disintegration at 82.34%. Texture showed a moderate level of predictability, with a mean accuracy of 72.38%, while color and odor achieved lower mean accuracies of 66.80% and 65.78%, respectively. Total score was the least predictable parameter, with a mean accuracy of 45.64%. These results indicate that purchasability, water release, and disintegration were more reliably predicted from visual information, whereas total score, which represents a more complex and integrative quality assessment, was considerably more difficult to classify.

Figure 7 presents the confusion matrices of the best-performing classification models for each sensory quality parameter. Overall, the matrices show a clear concentration of predictions along the main diagonal, indicating that the selected models were generally successful in assigning samples to their correct classes. The strongest diagonal patterns were observed for purchasability, water release, and disintegration, which is consistent with the high classification accuracies obtained for these parameters. Texture, color, and odor also demonstrated acceptable class-level discrimination, although the presence of several off-diagonal values indicates confusion among some neighboring classes. In contrast, total score exhibited the greatest degree of misclassification, with predictions distributed across multiple classes and a less distinct diagonal structure. These findings support the quantitative results reported in Table 4 and further suggest that sensory attributes associated with more direct and visually distinguishable changes were classified more reliably than more complex and integrative quality parameters.

To provide a class-wise interpretation of the image regions contributing to the classification decisions, a representative image set was selected to include one sample from each class of every sensory quality parameter. Grad-CAM analysis was then applied using the best-performing model identified for each parameter to visualize the spatial regions that contributed most strongly to the predicted class. This analysis was intended to examine whether the models primarily relied on information located on the fish surface or whether potentially confounding regions, such as specimen boundaries and the surrounding background, also contributed to the predictions. For each representative sample, the original image, Grad-CAM heatmap, Grad-CAM overlay, predicted class, and corresponding prediction confidence are presented in Table 5.

As shown in Table 5, the Grad-CAM visualizations generally indicated that the best-performing models concentrated their strongest activations on, or in close proximity to, the fish specimen rather than distributing attention uniformly across the entire image. Particularly coherent activation patterns were observed for several correctly classified samples, including no water leakage, firm texture, fresh and neutral odor, characteristic color, absence of disintegration, and the edible class. These samples were also associated with very high prediction confidence, reaching 0.9996 for no water leakage, 0.9998 for firm texture, 0.9945 for fresh and neutral odor, 0.9969 for characteristic color, 1.0000 for absence of disintegration, and 0.9983 for purchasability. Visual inspection of the overlays further showed that, for these relatively well-recognized classes, the highly activated regions frequently covered substantial portions of the fillet surface. In contrast, some more difficult classes exhibited more localized or fragmented activation patterns, with salient regions extending toward the specimen boundaries or adjacent background areas. This behavior was particularly evident for the seaweed-like odor sample, which was misclassified as sour-milk odor with a confidence of 0.4722, suggesting that the visual cues available for separating these odor-related classes were less distinctive.

The distinction was even more pronounced for total score. Although the representative samples for scores 0 and 1 were correctly classified, the samples corresponding to scores 2–9 were assigned to different score categories, generally with low confidence values. The corresponding Grad-CAM maps also showed comparatively heterogeneous spatial patterns: some activations were distributed over the specimen, whereas others were concentrated near its margins or extended into neighboring image regions. This variability is consistent with the lower classification performance obtained for total score and suggests that this integrative quality measure is not represented by a single, consistently localized visual feature. By comparison, individual attributes such as water release, texture, color, disintegration, and purchasability generally produced more coherent specimen-associated activation patterns in the representative examples. Overall, the Grad-CAM results indicate that the models predominantly exploited visual information associated with the fish specimen, while the more diffuse patterns observed for difficult classes also suggest that edge- or background-related cues may contribute to some decisions. These visualizations therefore provide spatial evidence for interpreting model behavior, but they should not be regarded as direct evidence that the networks detected specific biochemical mechanisms associated with spoilage.

### 3.2. Color Analysis Prediction Performance

Table 6 presents the performance of the regression models developed for the prediction of the mean L*, a*, and b* values. The models were ranked separately for each parameter according to their MAE values. Regression MAE values are reported as mean ± SD across the 10 fillet-level test folds. In addition, predictions were categorized as excellent, good, medium, or rejected based on the predefined absolute-error tolerances. The acceptable prediction rate represents the combined percentage of predictions classified as excellent, good, or medium.

For the prediction of a*, DenseNet121 achieved the lowest MAE of 0.680 and the highest acceptable prediction rate of 50.70%. MobileNet and VGG16 followed with MAE values of 0.718 and 0.764, respectively, while their acceptable prediction rates were 48.37% and 48.14%. InceptionV3 produced the highest MAE for a* prediction, at 0.820, and the lowest acceptable prediction rate, at 43.26%.

For b*, MobileNet achieved the lowest MAE of 1.200, with an acceptable prediction rate of 29.30%. DenseNet121 followed closely with an MAE of 1.206 and showed the highest acceptable prediction rate of 29.77%, although 70.23% of its predictions remained outside the predefined tolerance range. The other architectures produced acceptable prediction rates below 27%, with VGG19 showing the poorest performance, as indicated by an MAE of 1.716 and a rejection rate of 76.98%.

The prediction of L* remained more challenging than that of a*. DenseNet121 achieved the lowest MAE for L*, at 2.467, followed by MobileNet at 2.593 and InceptionV3 at 3.324. DenseNet121 also produced the highest acceptable prediction rate for L*, at 26.05%, while MobileNet reached 24.65%. VGG19 exhibited the poorest performance, with an MAE of 4.038 and a rejection rate of 87.91%. These findings demonstrate that the regression models were substantially more successful in predicting a* than either b* or L* under the specified tolerance criteria.

Despite these results, the tolerance-based evaluation indicates an important limitation of the regression models. Although DenseNet121 achieved an acceptable prediction rate of 50.70% for a*, the highest acceptable rates for b* and L* were only 29.77% and 26.05%, respectively. Therefore, the relatively low MAE values obtained for some parameters should not be interpreted as evidence that all color attributes can be predicted with sufficient precision to replace instrumental color measurements. Rather, the results indicate that prediction performance was strongly parameter-dependent and that small numerical variations, particularly in L* and b*, remained difficult to estimate reliably from RGB images. Image-based color estimation may also be affected by sample geometry, surface characteristics, and interactions between illumination and fillet thickness. Wang et al. [31], for example, reported parameter-specific effects of fillet thickness and illumination on image-based color assessment of large yellow croaker fillets. Consequently, the present regression models should be considered a non-destructive estimation approach rather than a direct substitute for instrumental color measurement.

Table 7 summarizes the MAE values obtained for the prediction of chroma, hue, and whiteness. MobileNet achieved the best performance for chroma, with the lowest MAE of 1.024, followed closely by InceptionV3 and DenseNet121, with MAE values of 1.053 and 1.054, respectively. For hue prediction, DenseNet121 produced the lowest MAE of 0.961. VGG19 and VGG16 ranked second and third, with very similar MAE values of 0.967 and 0.968, respectively, indicating relatively small performance differences among the top-performing models.

For whiteness, DenseNet121 achieved the lowest MAE of 2.357, followed by MobileNet and InceptionV3, with MAE values of 2.585 and 3.221, respectively. VGG16 and VGG19 showed comparatively weaker performance for whiteness, with MAE values of 3.926 and 4.006, respectively. Overall, the results indicate that the best-performing architecture varied depending on the derived color parameter, with MobileNet providing the lowest prediction error for chroma and DenseNet121 achieving the best performance for both hue and whiteness.

All regression models are considered together and have been visualized by heatmap presented in Figure 8 where lower values indicate more accurate predictions. Overall, DenseNet121 demonstrated the best regression performance, with a mean MAE of 1.428 across the six color parameters. MobileNet ranked second, with an overall mean MAE of 1.492, followed by InceptionV3 at 1.596. In contrast, VGG16 and VGG19 produced considerably higher overall MAE values of 2.268 and 2.283, respectively.

All regression models are considered together and visualized in the heatmap presented in Figure 8, where lower MAE values indicate more accurate predictions. Overall, DenseNet121 demonstrated the best regression performance, with a mean MAE of 1.454 across the six color parameters. MobileNet ranked second, with an overall mean MAE of 1.519, followed by InceptionV3 at 1.803. In contrast, VGG16 and VGG19 produced higher overall mean MAE values of 2.057 and 2.129, respectively.

No single architecture achieved the best result for every target parameter. DenseNet121 was the best-performing architecture for a*, L*, hue, and whiteness, with MAE values of 0.680, 2.467, 0.961, and 2.357, respectively. MobileNet achieved the lowest prediction errors for b* and chroma, with MAE values of 1.200 and 1.024, respectively. Although the differences among some architectures were relatively small for certain parameters, particularly hue and chroma, the heatmap indicates that DenseNet121 and MobileNet provided the most consistent overall regression performance. InceptionV3 showed intermediate performance, whereas VGG16 and VGG19 generally produced higher prediction errors, particularly for L* and whiteness.

Figure 9 compares the best, mean, and worst MAE values obtained for each color parameter across all transfer-learning architectures. Based on the mean MAE values, a* was the most accurately predicted parameter, with an average MAE of 0.756. Hue and chroma followed, with average MAE values of 0.979 and 1.108, respectively. The mean MAE obtained for b* was 1.406, whereas whiteness and L* produced considerably higher values of 3.219 and 3.287, respectively.

The difference between the best- and worst-performing architectures was smallest for hue, with an MAE range of only 0.042, followed by a* with a range of 0.140. The relatively narrow ranges observed for these parameters indicate that their prediction performance was comparatively stable across the evaluated architectures. Chroma and b* showed moderately larger ranges of 0.226 and 0.516, respectively. In contrast, the ranges observed for whiteness and L* were substantially larger, at approximately 1.649 and 1.571, respectively, indicating that the choice of transfer-learning architecture had a considerably greater influence on the prediction accuracy of these parameters.

### 3.3. Overall Ranking of Transfer-Learning Architectures

Figure 10 presents the rank distribution of the five transfer-learning architectures across all 13 target parameters, comprising seven classification and six regression tasks. DenseNet121 demonstrated the strongest overall performance, ranking first for nine parameters, second for three parameters, and third for one parameter. Notably, it was never ranked fourth or fifth, indicating consistently strong performance across the evaluated prediction tasks. MobileNet also showed strong overall performance, achieving first place for four parameters and second place for eight parameters. It ranked fourth for only one parameter and was never ranked third or fifth.

InceptionV3 exhibited intermediate performance, ranking second for one parameter and third for nine parameters. It also ranked fourth for one parameter and fifth for two parameters, while it did not achieve a first-place ranking. In contrast, the VGG-based architectures generally occupied the lower ranks. VGG16 ranked third for three parameters and fourth for the remaining ten parameters, without achieving either a first- or second-place ranking. VGG19 showed the weakest overall performance, ranking fifth for 11 parameters, while ranking second and fourth for one parameter each. Overall, the rank distribution clearly indicates that DenseNet121 provided the most consistently superior performance, followed by MobileNet, whereas VGG16 and particularly VGG19 were predominantly associated with the lower performance ranks.

Overall, the ranking distribution indicates that DenseNet121 provided the highest number of best-performing results, while MobileNet and InceptionV3 offered strong and relatively consistent alternatives. The VGG-based architectures were generally less effective for the image-based prediction of fillet quality parameters.

### 3.4. Robustness Analysis Under Simulated Industrial Imaging Variations

To evaluate the sensitivity of the developed models to potential deviations from the controlled image-acquisition conditions, an additional robustness analysis was conducted using representative images and the best-performing model identified for each target parameter. The models were not retrained during this analysis. For the classification tasks, five images were selected from each class of the seven sensory quality parameters to provide balanced class representation. For the regression tasks, five representative images were selected for each of the six color parameters. Rather than selecting regression samples randomly, the images were chosen to approximately represent the lower, lower-middle, middle, upper-middle, and upper portions of the observed value distribution, corresponding approximately to the 10th, 30th, 50th, 70th, and 90th percentiles. This selection strategy was used to examine model sensitivity across a broad range of target values.

Each selected image was first evaluated in its original form and subsequently subjected to five controlled image perturbations designed to simulate sources of variability that may occur under industrial imaging conditions. Two illumination scenarios were generated by decreasing and increasing image brightness by 20%, respectively. Positional variability was simulated by rotating the images by 10°. Condensation or reduced optical clarity was approximated by applying Gaussian blur together with a low-intensity white haze layer. In addition, specular glare and reflection, which may occur due to transparent packaging or industrial illumination, were simulated by introducing semi-transparent and spatially smoothed bright regions. Representative examples of the original and perturbed image conditions, generated from the same fillet sample, are presented in Figure 11.

Thus, each selected image was evaluated under six conditions: the original image and five perturbed versions. All images were processed using the same input dimensions and normalization procedure as those used during model development. For the classification tasks, model robustness under each imaging condition was evaluated using classification accuracy, whereas for the regression tasks, robustness was assessed using mean absolute error (MAE). These metrics were calculated separately for the original and perturbed image conditions to quantify changes in predictive performance associated with each simulated imaging variation.

Table 8 summarizes the robustness of the best-performing classification and regression models under the simulated imaging variations. For the classification tasks, the response to image perturbations varied according to the target parameter. Disintegration classification was the most stable, maintaining an accuracy of 0.800 under all tested conditions. Purchasability also showed relatively high robustness, with accuracy remaining between 0.700 and 0.900 across the perturbation scenarios. Texture classification was unaffected by brightness changes, retaining the original accuracy of 0.867 under both low- and high-brightness conditions, whereas its performance decreased under rotation and condensation/blur to 0.600 and 0.400, respectively. Color classification was similarly sensitive to condensation/blur, with accuracy decreasing from 0.800 for the original images to 0.467, while rotation resulted in a slightly higher accuracy of 0.867. Odor classification showed comparatively limited variation across conditions, with accuracy ranging from 0.650 to 0.750. Water release exhibited greater variability, with accuracy decreasing from 0.600 to 0.400 under low brightness but increasing to 0.900 under both high brightness and glare/reflection. Total Score remained the most difficult classification target, with an original accuracy of 0.280 and further decreases under rotation and condensation/blur to 0.160 and 0.100, respectively. Overall, condensation/blur produced the most pronounced reductions in classification performance for several sensory attributes, particularly texture, color, Total Score, and purchasability.

The regression models also exhibited parameter-dependent sensitivity to the simulated imaging variations. The prediction of a* was comparatively stable across all conditions, with MAE values ranging from 0.551 to 0.663 compared with 0.620 for the original images. Chroma and hue also showed relatively moderate variations, although rotation increased the MAE to 0.863 for chroma and 0.865 for hue. For b*, the highest prediction error was observed under glare/reflection, where MAE increased from 0.975 for the original images to 1.409. The strongest sensitivity was observed for L* and whiteness under the condensation/blur condition. The MAE for L* increased markedly from 1.860 to 10.907, while that for whiteness increased from 2.352 to 10.819. In contrast, the errors for these parameters remained considerably lower under the other simulated conditions. These findings indicate that the robustness of the regression models depended strongly on both the predicted color attribute and the type of image degradation, with lightness-related parameters being particularly sensitive to condensation- and blur-like visual degradation.

Since the robustness analysis was performed on a deliberately selected representative subset, the values in Table 8 represent subset-specific baselines and should not be directly compared with the 10-fold cross-validation metrics reported in Table 4, Table 6 and Table 7.

## 4. Conclusions

This study demonstrated the potential of deep learning–based image analysis for estimating the freshness and quality of European seabass fillets from visually observable characteristics. Seven sensory quality parameters and six color attributes were evaluated, with purchasability regarded as the principal indicator of practical freshness. A total of 65 independently trained models, consisting of 35 classification and 30 regression models based on DenseNet121, InceptionV3, MobileNet, VGG16, and VGG19, were evaluated using group-based 10-fold cross-validation, in which each fillet served once as an independent test group. This data-partitioning strategy prevented images originating from the same fillet from being simultaneously included in the training and test sets and therefore provided a more rigorous assessment of model generalization.

The classification results showed that model performance depended on both the selected architecture and the target quality parameter. DenseNet121 achieved the highest accuracy for water release, odor, color, disintegration, and purchasability, with accuracies of 90.20%, 75.60%, 77.40%, 87.70%, and 92.60%, respectively. MobileNet achieved the best results for texture and total-score classification, with accuracies of 81.20% and 57.70%, respectively. Among all architecture–parameter combinations, the highest overall classification performance was obtained by DenseNet121 for purchasability, which also achieved a precision of 0.914, recall of 0.899, and F1-score of 0.891. In contrast, total score remained the most difficult classification target, with even the best-performing model achieving an F1-score of only 0.304. DenseNet121 also showed the highest mean classification accuracy across the seven sensory parameters at 79.30%, followed by MobileNet at 78.40% and InceptionV3 at 72.80%.

When predictability was evaluated across architectures, purchasability was the most reliably classified sensory parameter, with a mean accuracy of 86.84%, followed by water release at 84.94% and disintegration at 82.34%. Texture showed intermediate predictability at 72.38%, whereas color and odor reached mean accuracies of 66.80% and 65.78%, respectively. Total score was markedly more difficult to predict, with a mean accuracy of only 45.64%. These findings indicate that sensory attributes associated more directly with visually observable changes in the fillet can be estimated more successfully from RGB images, whereas complex or partly non-visual attributes remain more challenging. In particular, odor cannot be directly captured by an image, and total score integrates several sensory characteristics rather than representing a single localized visual feature.

The Grad-CAM analysis provided additional insight into the spatial basis of the classification decisions. For several correctly classified and highly confident samples, including no water leakage, firm texture, fresh and neutral odor, characteristic color, absence of disintegration, and purchasability, the strongest activations were predominantly associated with the fillet surface. In contrast, more difficult classes showed more localized or fragmented activation patterns, sometimes extending toward specimen boundaries or adjacent background regions. This pattern was particularly evident for odor and total-score prediction. Total-score samples also exhibited heterogeneous activation patterns and frequent class confusion, supporting the quantitative finding that this integrative parameter cannot be represented reliably by a single consistent visual feature. Accordingly, the Grad-CAM maps support the interpretation that the models predominantly used specimen-associated visual information, while also indicating that edge- or background-related cues may contribute to some difficult predictions. These visualizations should therefore be interpreted as spatial explanations of model decisions rather than as direct evidence of specific biochemical spoilage mechanisms.

The regression results similarly demonstrated parameter-dependent performance. DenseNet121 achieved the lowest MAE for a* (0.680), L* (2.467), hue (0.961), and whiteness (2.357), whereas MobileNet achieved the lowest MAE for b* (1.200) and chroma (1.024). Among the directly measured CIELab parameters, a* was predicted more successfully than b* and L* according to the predefined tolerance criteria, with DenseNet121 achieving an acceptable prediction rate of 50.70% for a*. In contrast, even the best-performing models achieved acceptable prediction rates of only 29.77% for b* and 26.05% for L*, indicating that small variations in these parameters remain difficult to estimate accurately from RGB images. Across all six regression targets, DenseNet121 achieved the lowest overall mean MAE of 1.454, followed by MobileNet at 1.519 and InceptionV3 at 1.803, whereas VGG16 and VGG19 produced higher overall mean MAE values of 2.057 and 2.129, respectively.

The overall ranking across all 13 prediction targets further confirmed the superiority of DenseNet121 and MobileNet. DenseNet121 ranked first for nine parameters, second for three, and third for one, and was never ranked fourth or fifth. MobileNet ranked first for four parameters and second for eight, while InceptionV3 showed predominantly intermediate performance. VGG16 and particularly VGG19 generally occupied the lower ranks. Thus, although no single architecture was optimal for every target, DenseNet121 demonstrated the strongest overall consistency, with MobileNet providing a competitive and computationally efficient alternative.

The robustness analysis further revealed parameter-dependent sensitivity to simulated imaging variations, with condensation/blur particularly affecting L* and whiteness prediction, whereas some classification targets, such as disintegration, remained comparatively stable.

Overall, the findings support the feasibility of using deep learning and conventional RGB imaging for non-destructive estimation of selected freshness and quality indicators of European seabass fillets under controlled imaging conditions. Nevertheless, further validation using larger and more heterogeneous datasets from different production batches, fish sizes, storage conditions, seasons, imaging devices, illumination environments, and processing facilities is required before industrial deployment. Future validation should continue to preserve fillet- or batch-level separation between training and evaluation data and should preferably include fully independent external datasets. Data augmentation, class-balancing strategies, partial fine-tuning of pretrained convolutional layers, systematic hyperparameter optimization, fillet segmentation, and color calibration may further improve generalization and reduce the influence of non-quality-related image variation. Finally, multimodal approaches integrating RGB images with complementary measurements such as temperature, storage duration, volatile compounds, pH, electronic-nose signals, or hyperspectral information may be particularly beneficial for difficult targets such as odor, total score, and early-stage spoilage. Such systems may ultimately support rapid and objective seafood quality control, automated purchasability assessment, reduced food waste, and improved management of refrigerated supply chains.

## Figures and Tables

**Figure 1:**
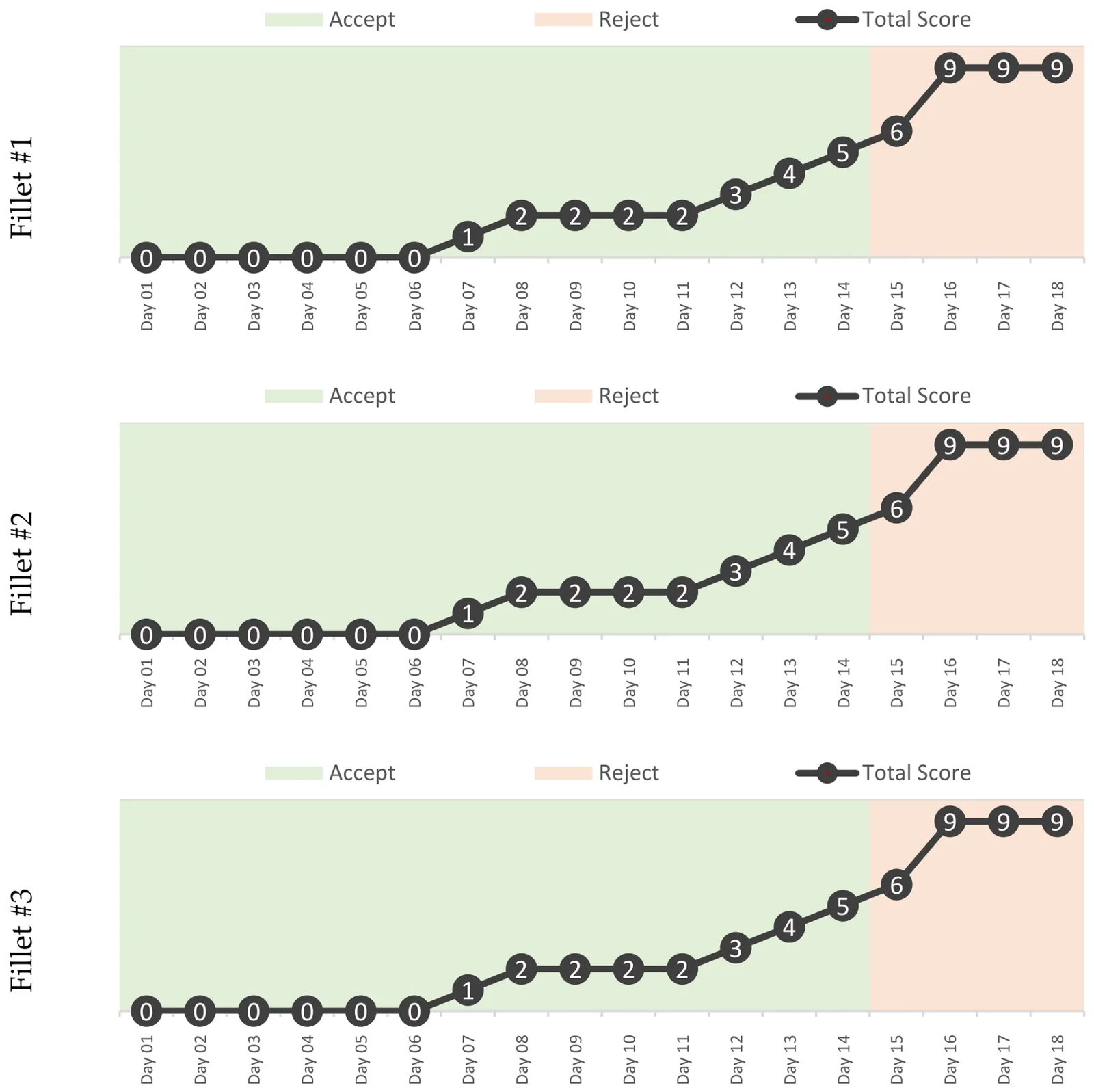

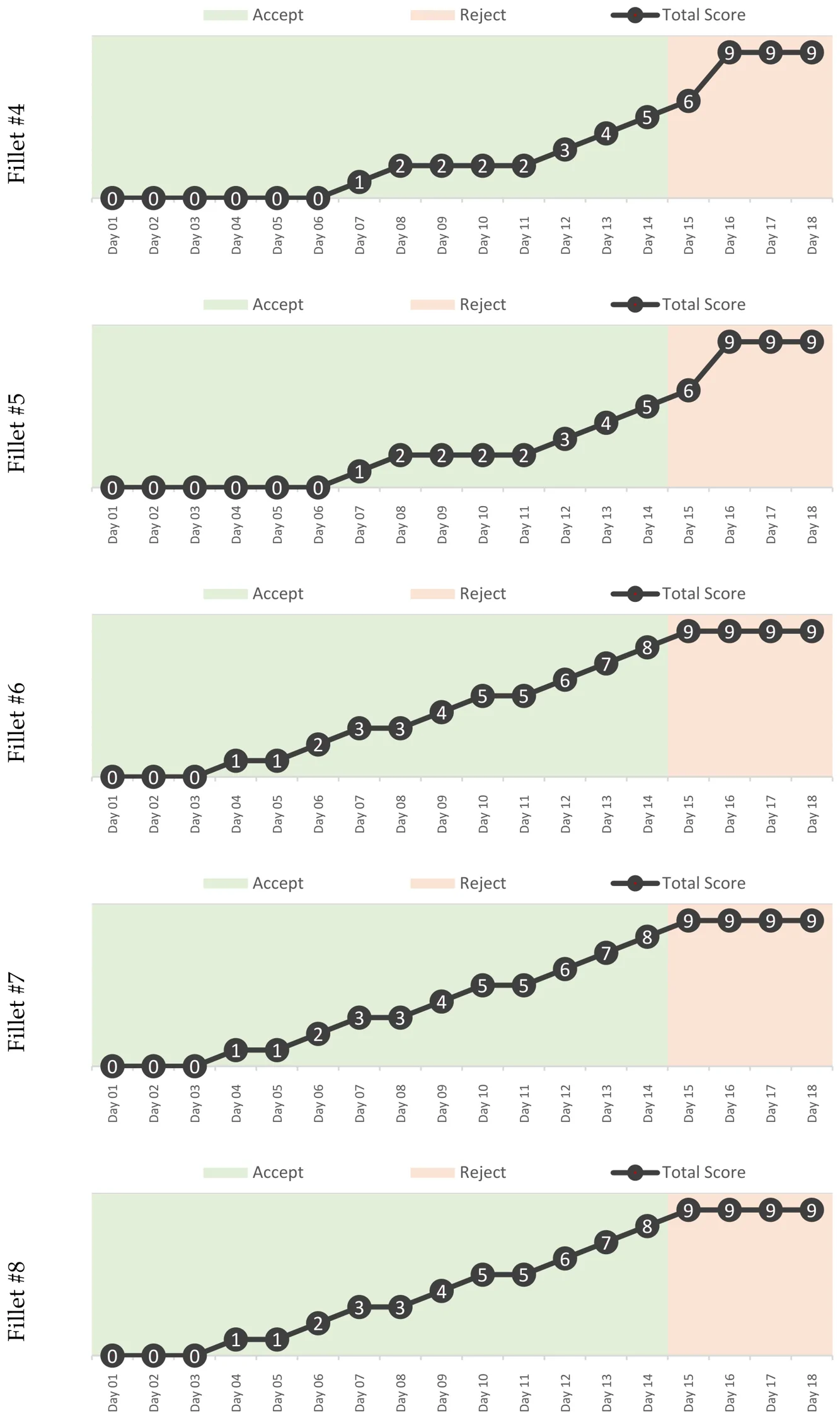

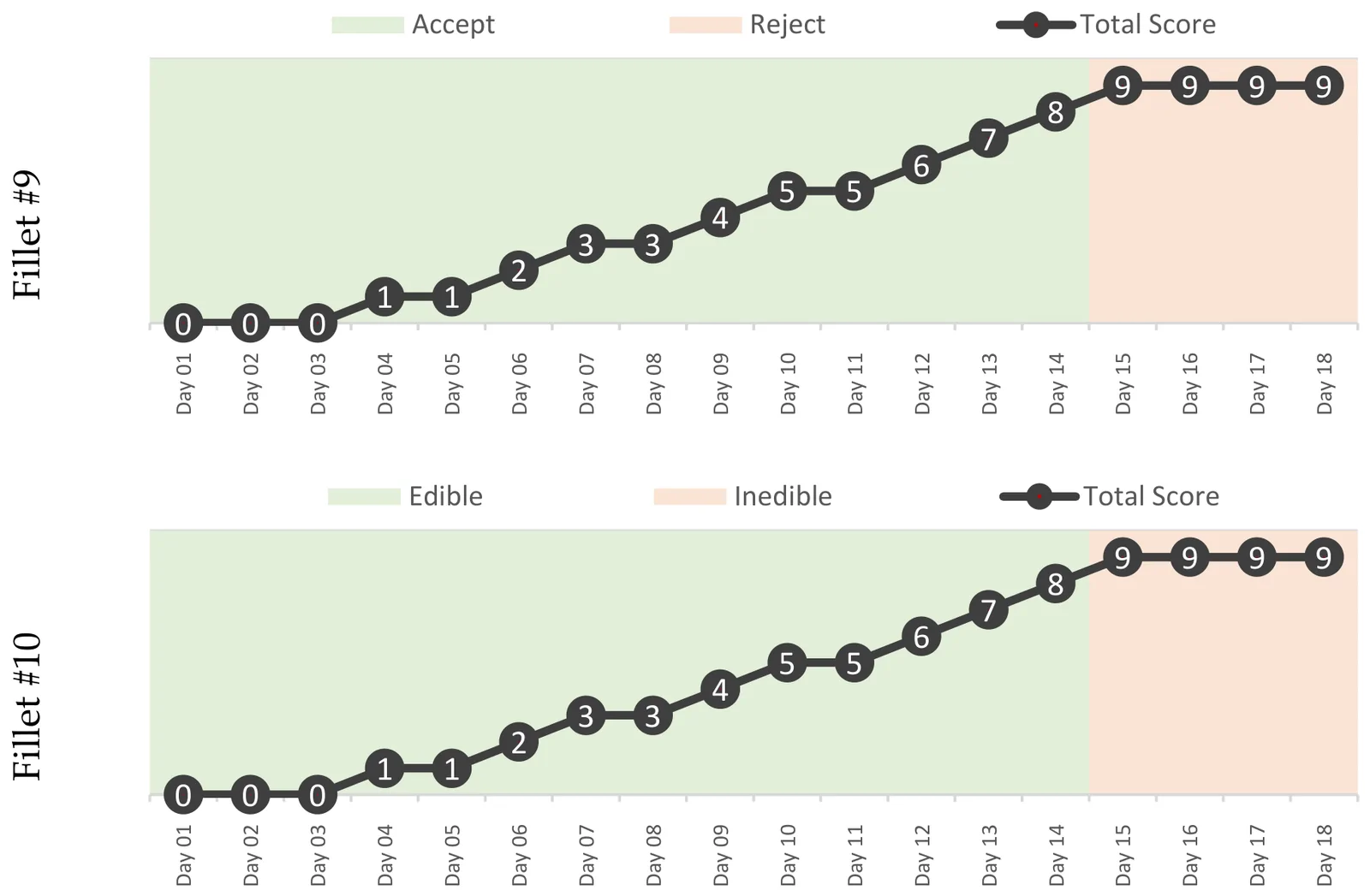
Comparison of the daily total quality scores and expert-assigned purchasability status of the 10 seabass fillet samples.

**Figure 2:**
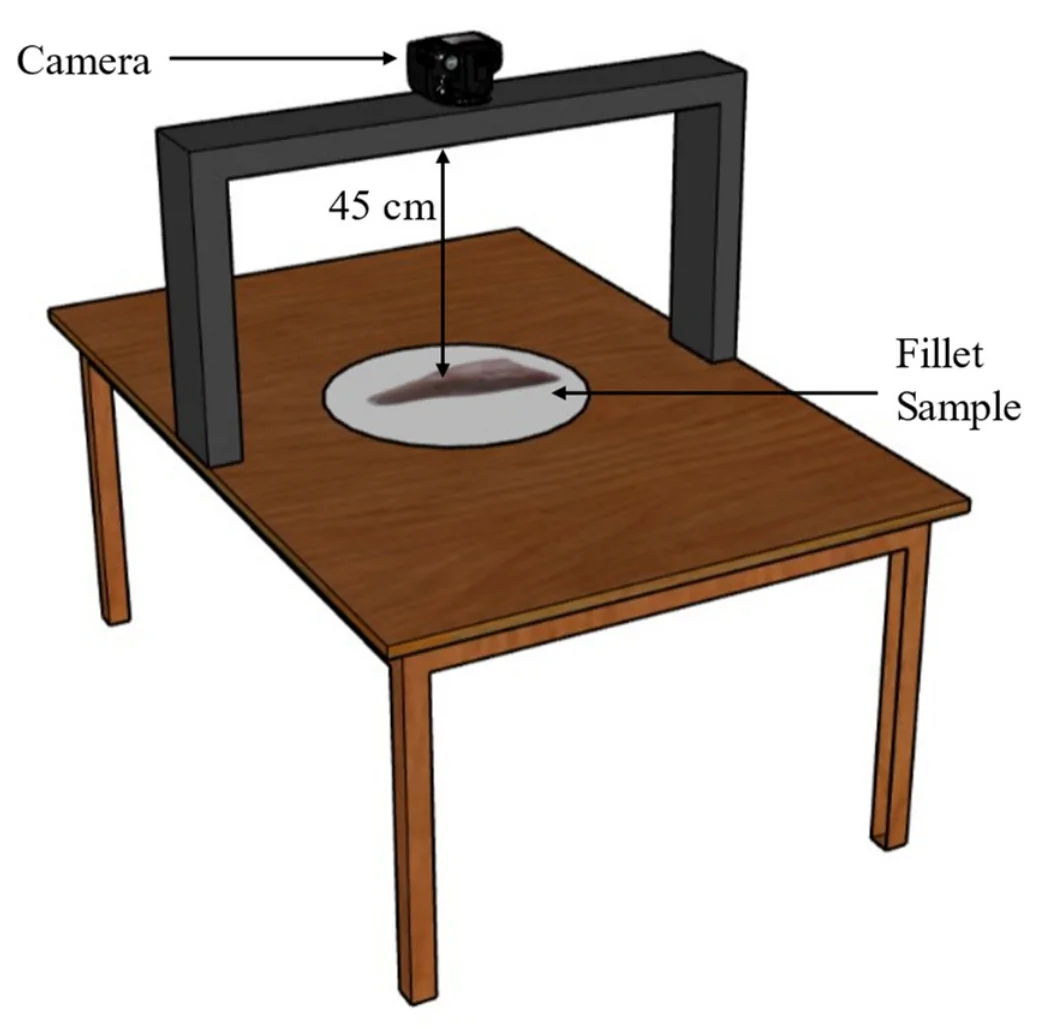
Schematic representation of the custom-built imaging stand used for image acquisition.

**Figure 3:**
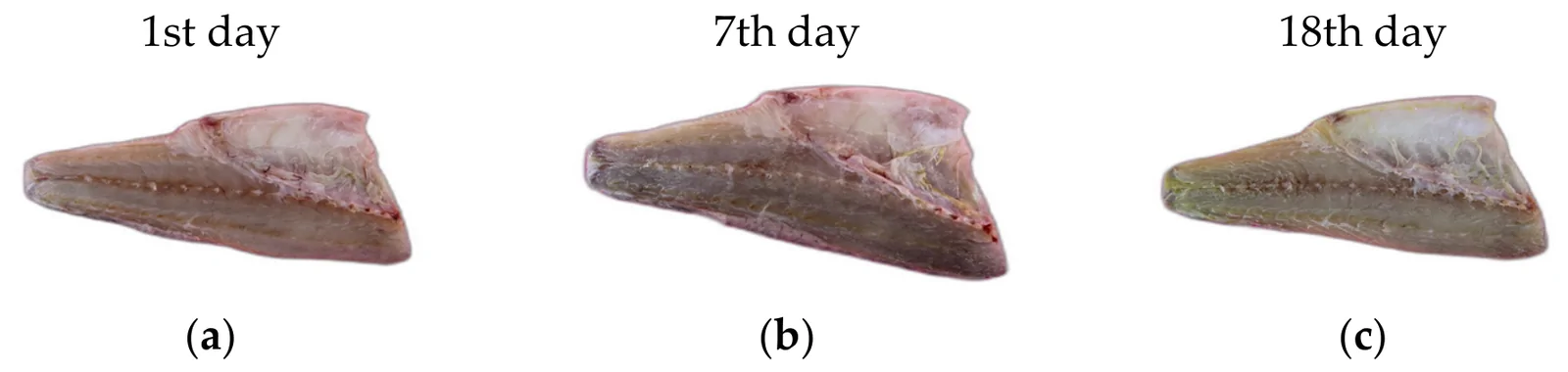
Representative images of the same seabass fillet acquired on three different storage days (**a**) 1st day (**b**) 7th day (**c**) 18th day, illustrating the progressive visual changes from the fresh state to an advanced stage of spoilage.

**Figure 4:**
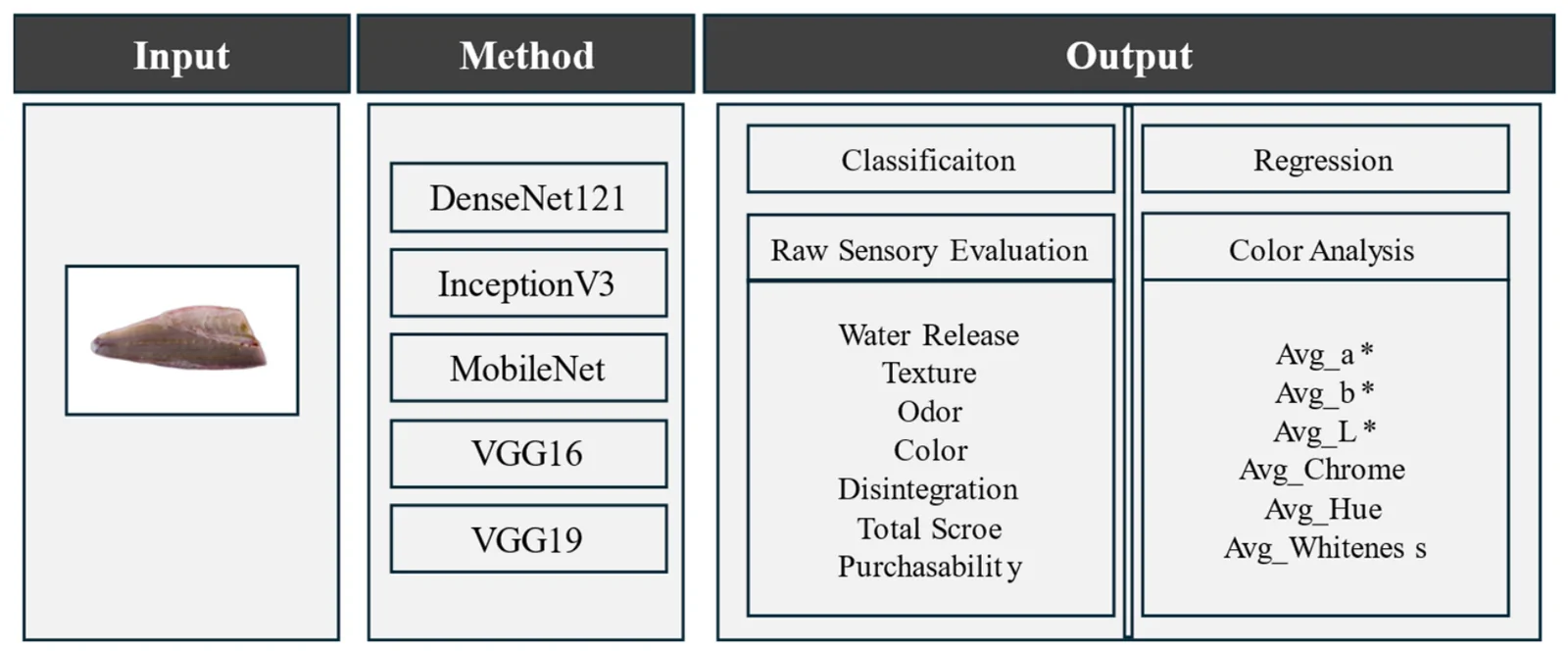
Overview of the proposed image-based methodology for predicting seabass fillet quality attributes.

**Figure 5:**
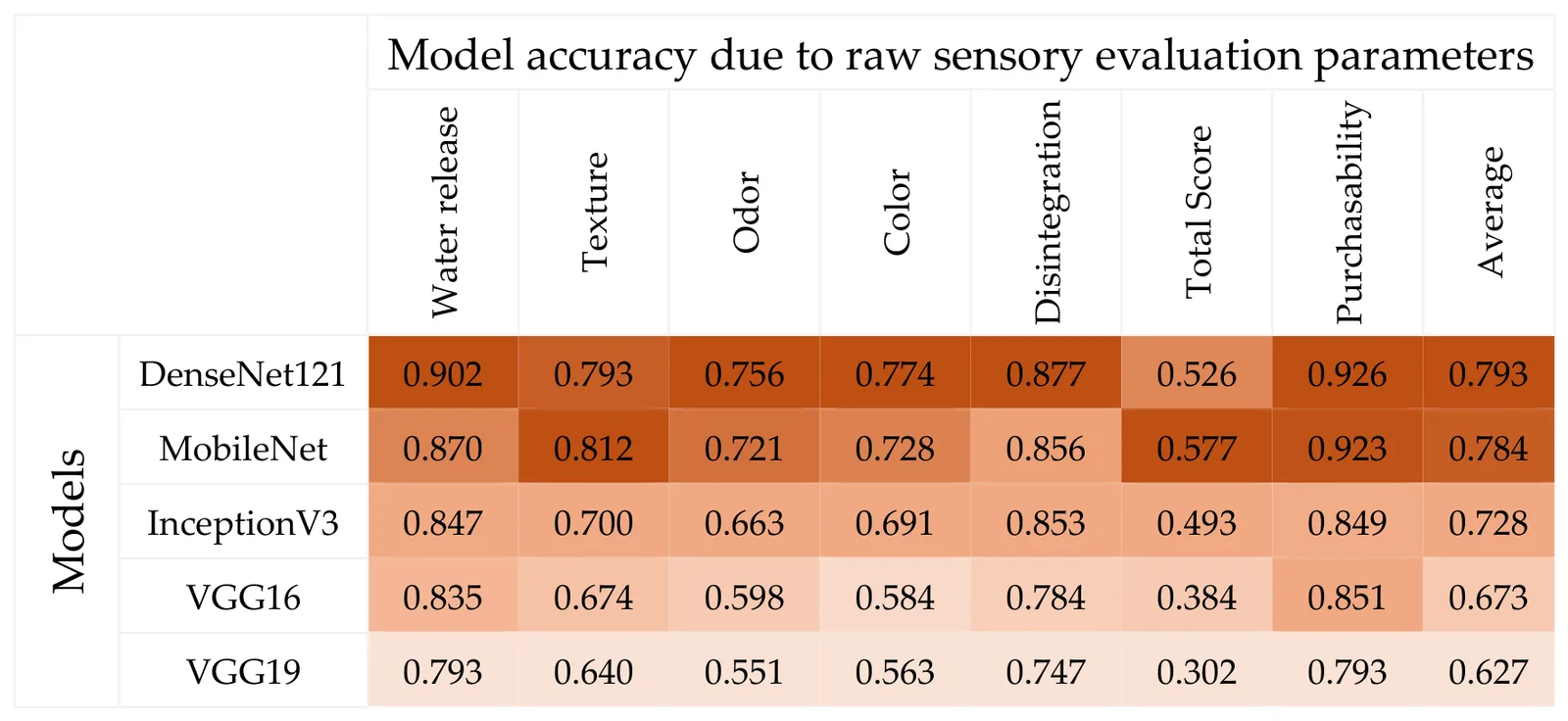
Heatmap of classification accuracy obtained for the five transfer-learning architectures across the seven sensory quality parameters. The color scale represents classification accuracy, with darker and lighter colors indicating higher and lower accuracy values, respectively.

**Figure 6:**
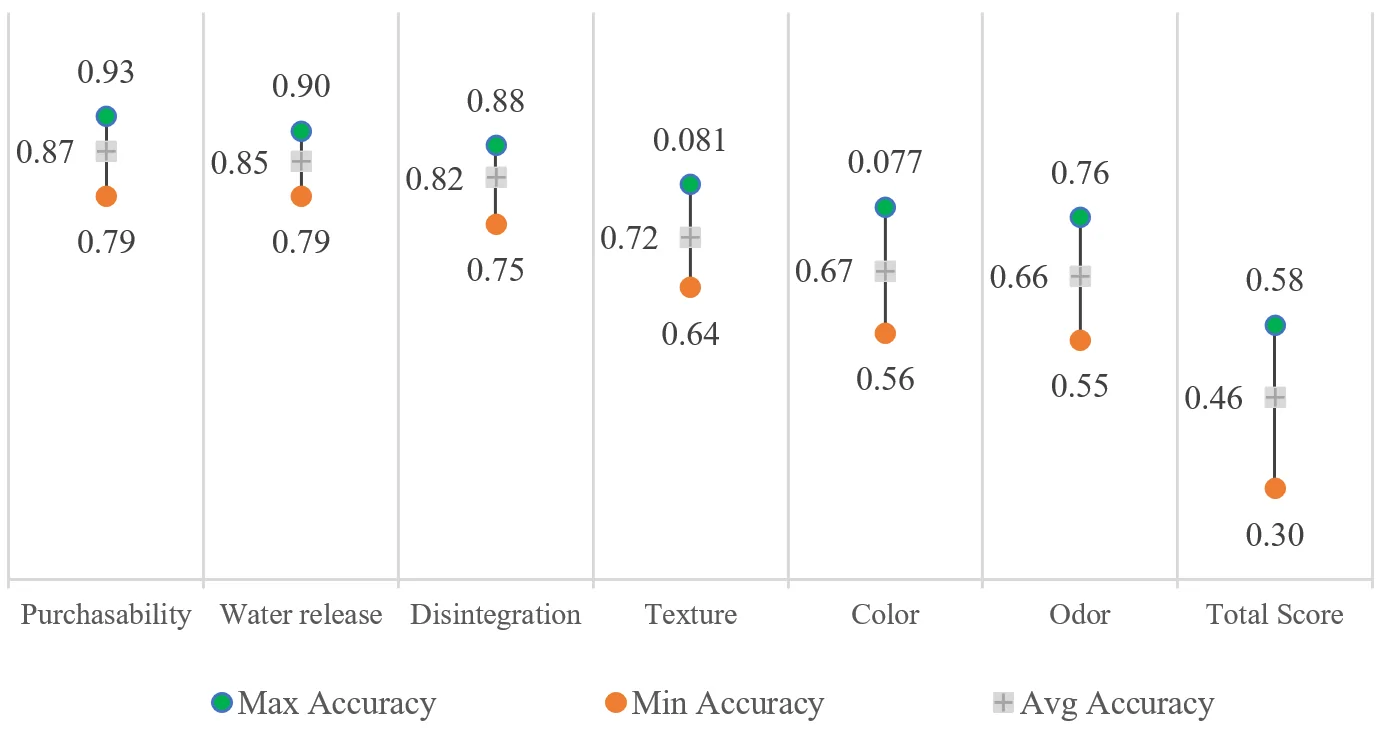
Predictability of raw sensory quality parameters.

**Figure 7:**
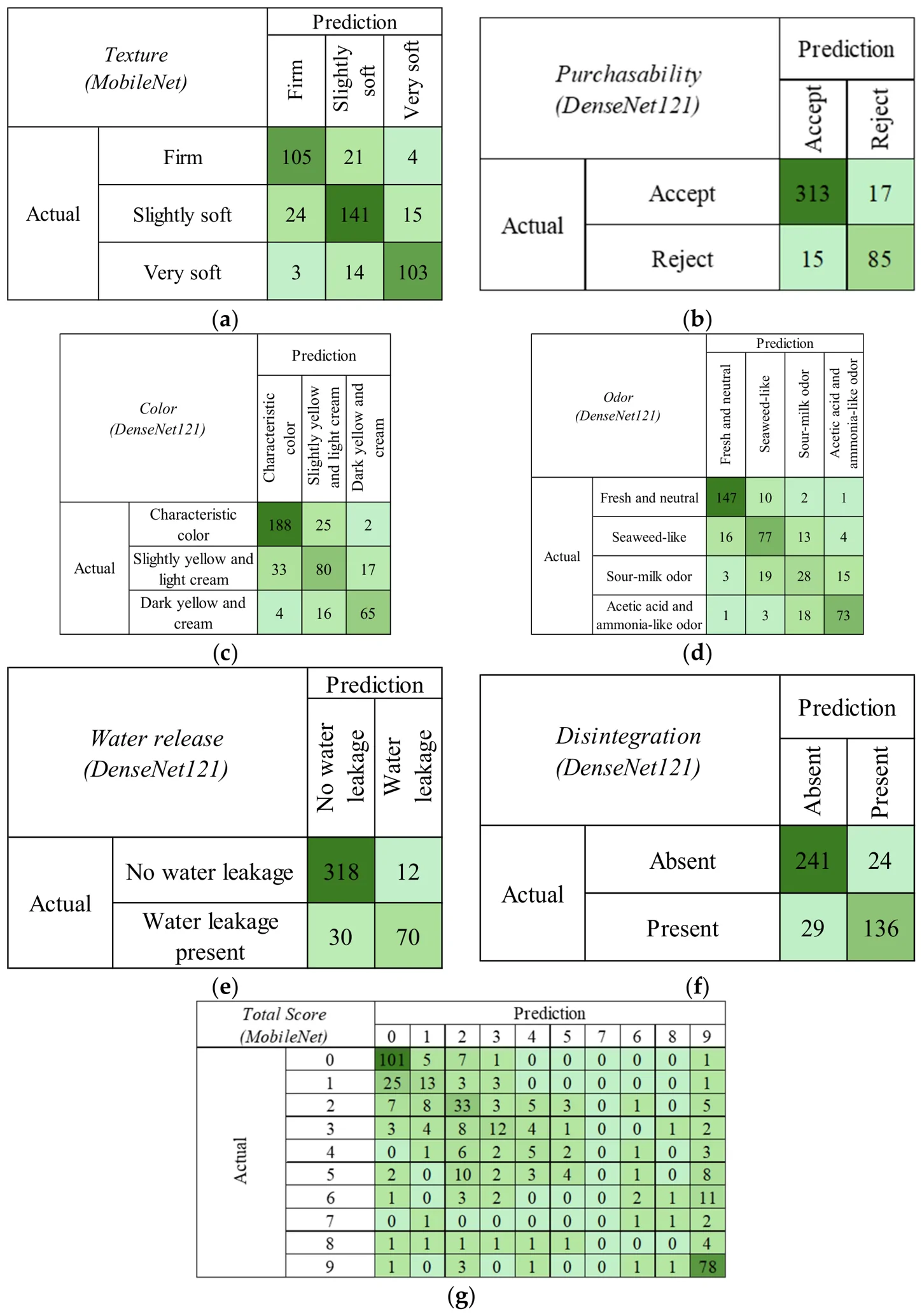
Confusion matrices obtained from the test predictions of the best-performing classification models for the sensory quality parameters. Rows represent the actual classes, whereas columns represent the predicted classes. The values within the cells indicate the number of test samples, and the color intensity represents the corresponding sample frequency, with darker colors indicating higher numbers of samples: (**a**) texture, (**b**) purchasability, (**c**) color, (**d**) odor, (**e**) water release, (**f**) disintegration, and (**g**) total score.

**Figure 8:**
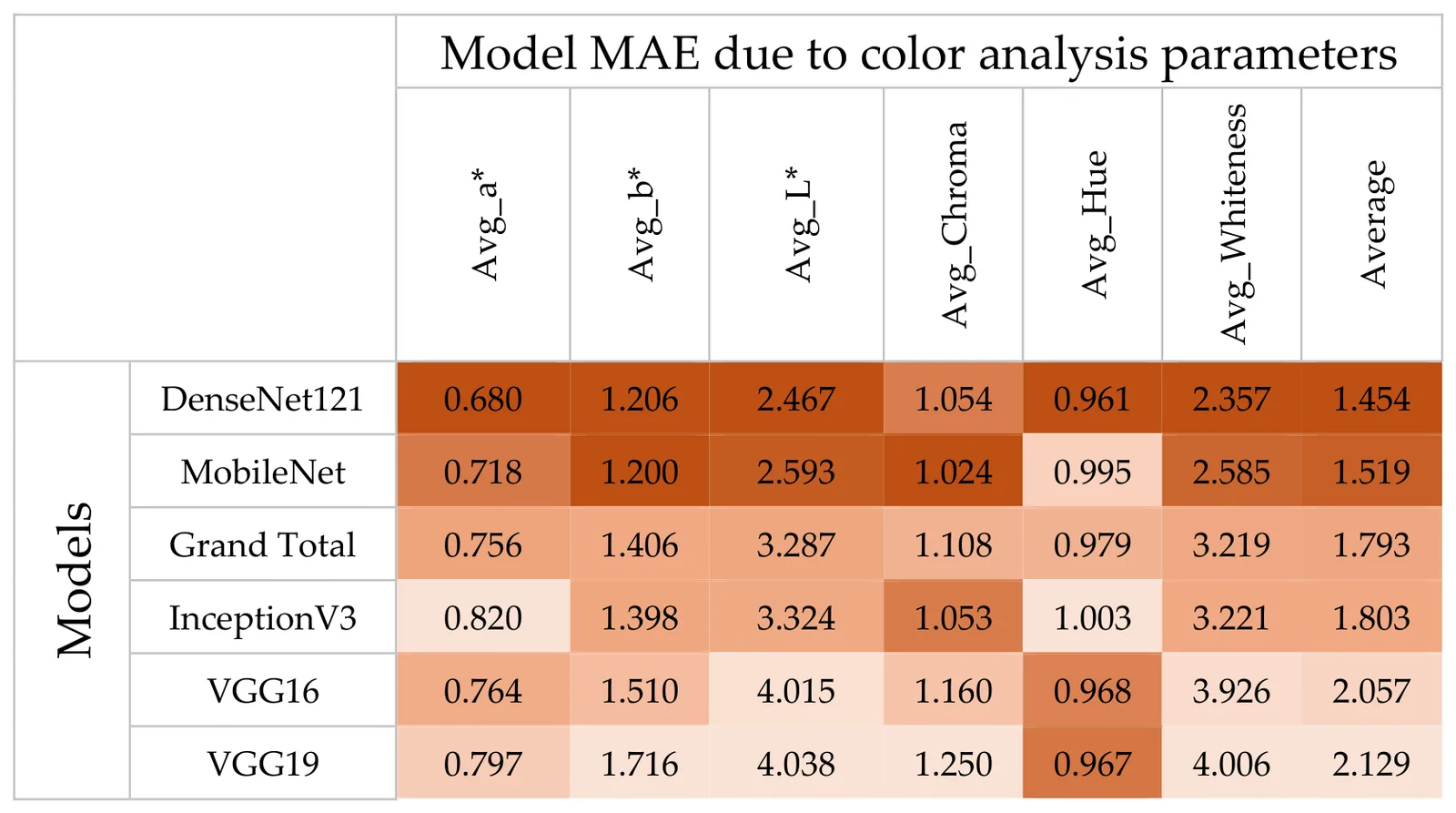
Heatmap of mean absolute error (MAE) obtained for the five transfer-learning architectures across the six color parameters. The values within the cells represent the corresponding MAE values. The color scale represents the magnitude of the prediction error, with lighter and darker colors indicating lower and higher MAE values, respectively.

**Figure 9:**
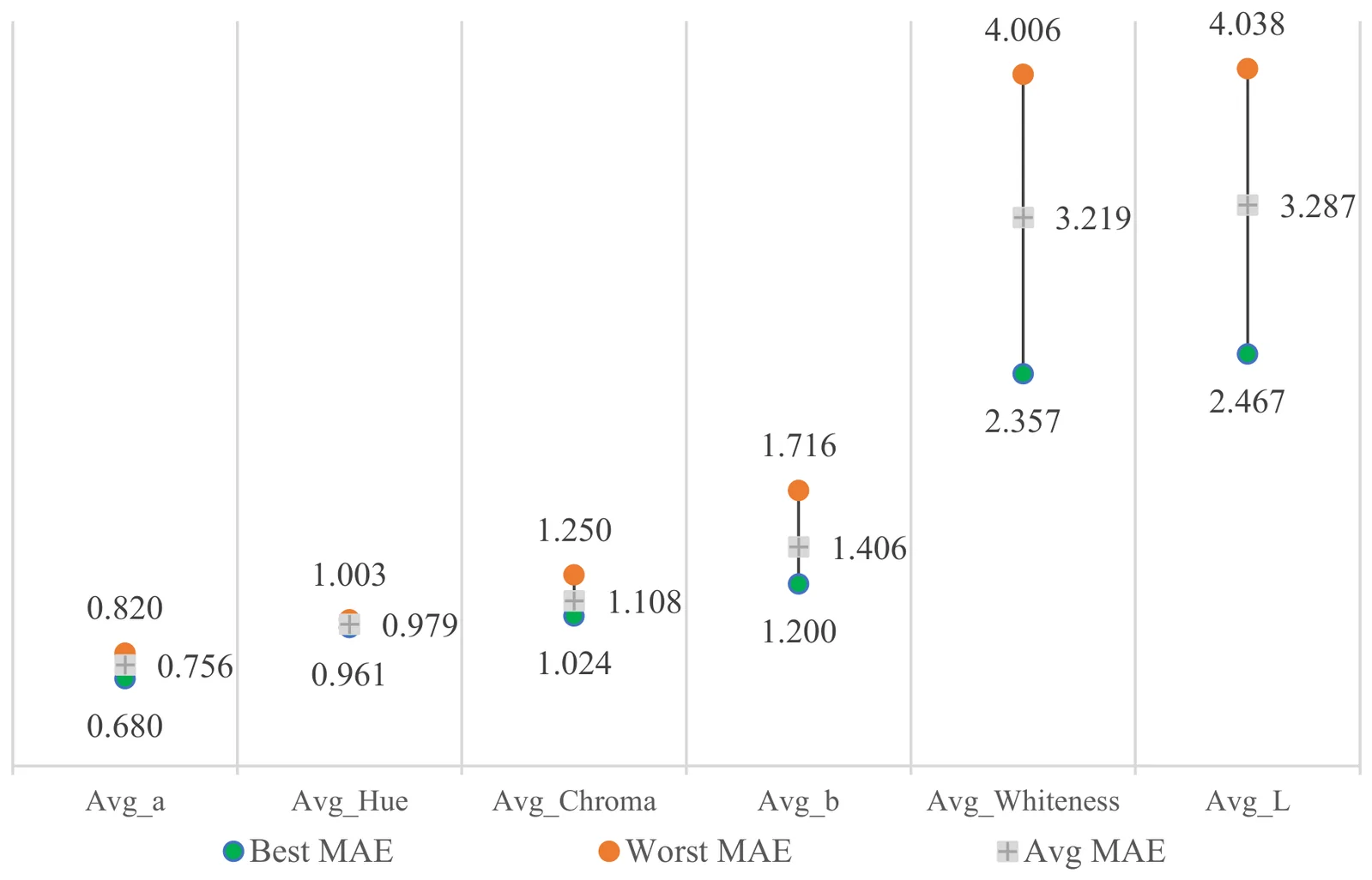
Predictability of color parameters.

**Figure 10:**
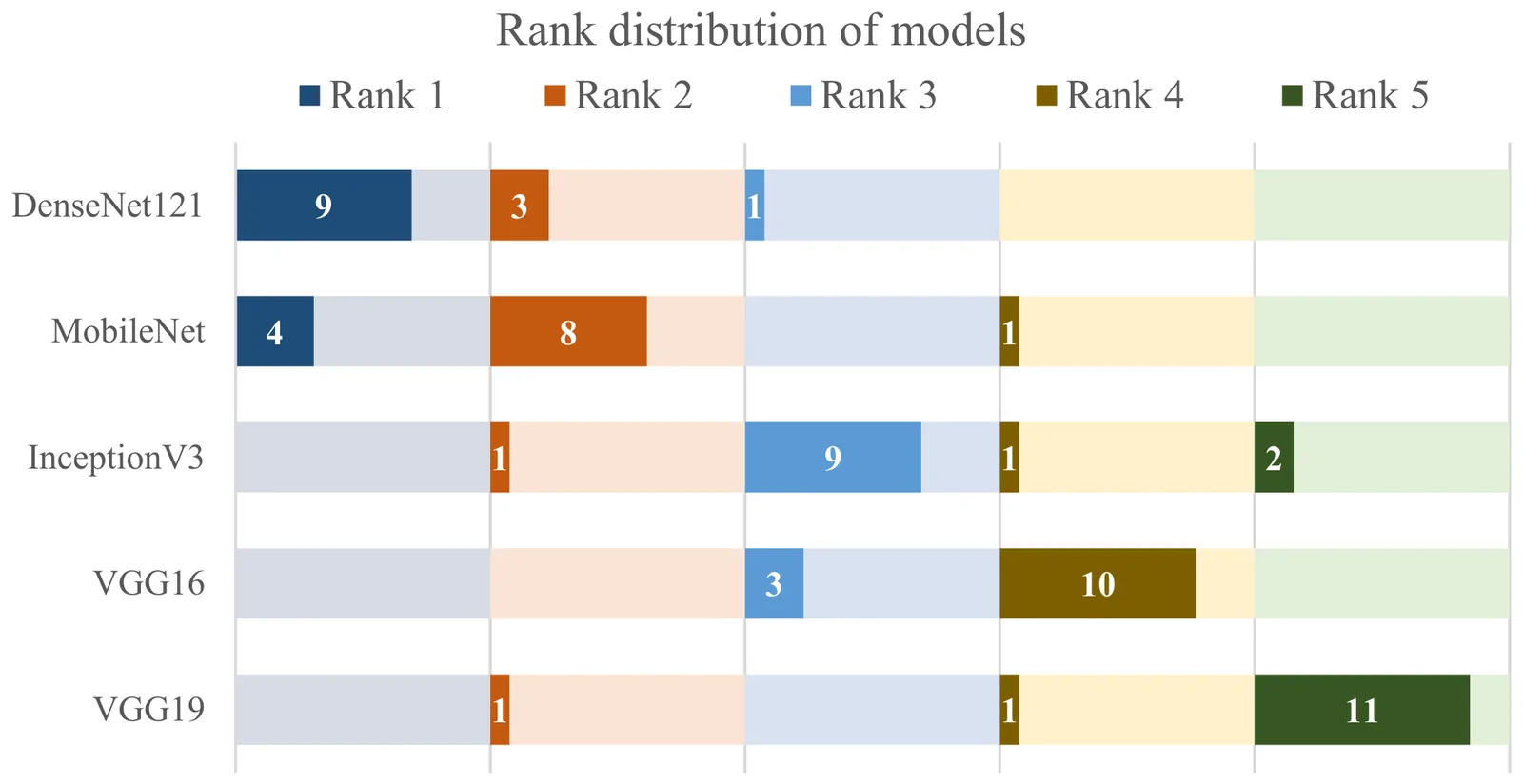
Distribution of the performance ranks achieved by all transfer-learning architectures across all parameters.

**Figure 11:**
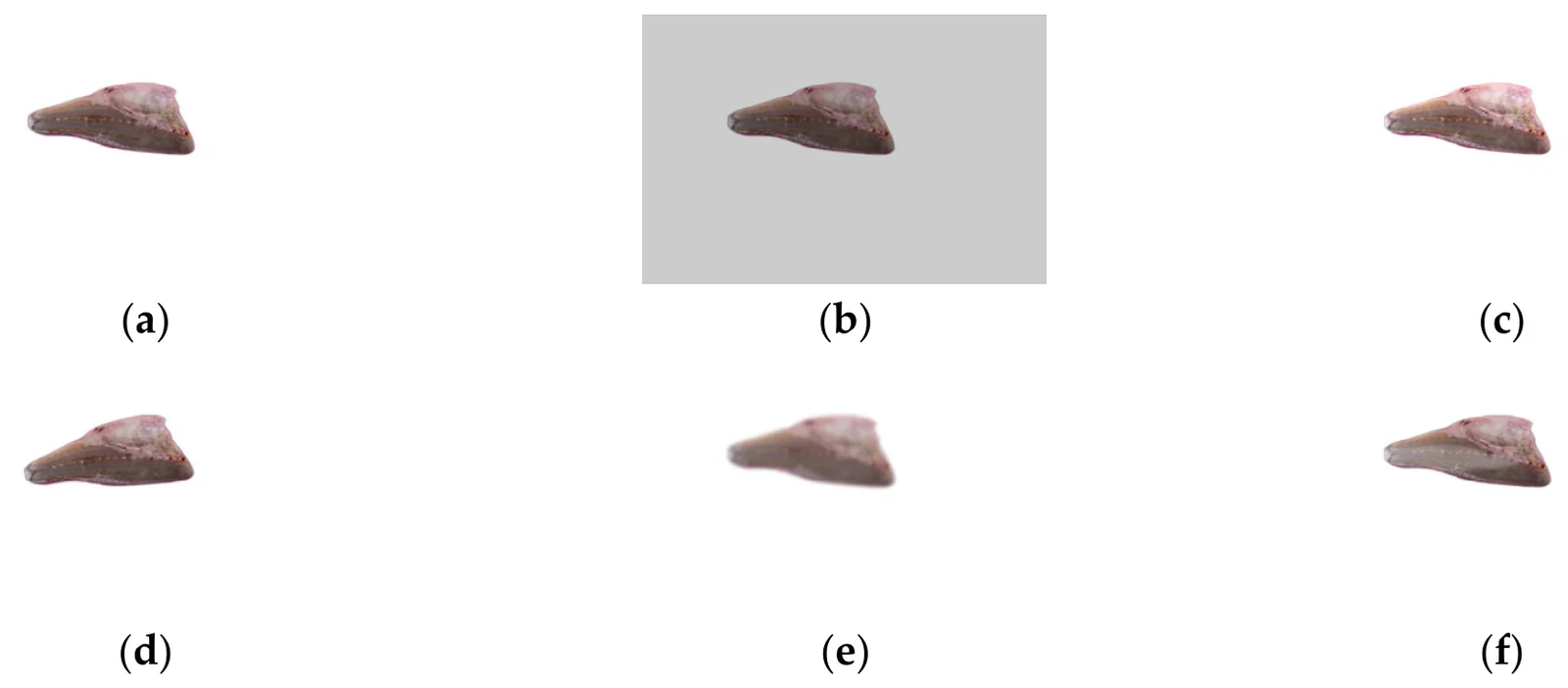
Representative example of the simulated imaging variations applied to a single fillet sample in the robustness analysis: (**a**) original image, (**b**) low brightness (−20%), (**c**) high brightness (+20%), (**d**) rotation (+10°), (**e**) condensation–blur, and (**f**) glare–reflection.

**Table 1:** Quality parameters and their evaluation criteria adapted from Bonilla et al. [18].

Quality Parameter	Score	Description
Water release	0	No water leakage
	1	Water leakage present
Texture	0	Firm
	1	Slightly soft
	2	Very soft
Odor	0	Fresh and neutral
	1	Seaweed-like
	2	Sour-milk odor
	3	Acetic acid and ammonia-like odor
Color	0	Characteristic color
	1	Slightly yellow and light cream
	2	Dark yellow and cream
Disintegration	0	Absent
	1	Present
Total Score	0–6	Not Spoiled
	7–9	Spoiled
Purchasability	1	Accept
	2	Reject

**Table 2:** Distribution of images among the classes for each sensory quality parameter.

Quality Parameter	Score	Description	Number of Images
Water release	0	No water leakage	330
	1	Water leakage present	100
Texture	0	Firm	130
	1	Slightly soft	180
	2	Very soft	120
Odor	0	Fresh and neutral	160
	1	Seaweed-like	110
	2	Sour-milk odor	65
	3	Acetic acid and ammonia-like odor	95
Color	0	Characteristic color	215
	1	Slightly yellow and light cream	130
	2	Dark yellow and cream	85
Disintegration	0	Absent	265
	1	Present	165
Total Score	0 (115), 1 (45), 2 (65), 3 (35), 4 (20), 5 (30), 6 (20), 7 (5), 8 (10), 9 (85)		
Purchasability	1	Accept	330
	2	Reject	100

**Table 3:** Architectural and training configurations of predictive models.

Model Parameters	Regression Models	Classification Models
Transfer-learning architectures	DenseNet121, InceptionV3, MobileNet, VGG16, and VGG19	DenseNet121, InceptionV3, MobileNet, VGG16, and VGG19
Pretrained weights	ImageNet	ImageNet
Input dimensions	224 × 224 × 3	224 × 224 × 3
CNN backbone	Frozen	Frozen
Pooling layer	Global average pooling	Global average pooling
Fully connected layer	64 neurons, ReLU activation	64 neurons, ReLU activation
Output layer	One neuron	Number of neurons corresponding to the number of classes
Output activation	Linear	SoftMax
Data partitioning	Group-based 10-fold cross-validation: 8 groups for training, 1 group for validation, and 1 group for testing in each fold	Group-based 10-fold cross-validation: 8 groups for training, 1 group for validation, and 1 group for testing in each fold
Random seed	42 + fold number for validation-group selection	42 + fold number for validation-group selection
Optimizer	Adam	Adam
Loss function	Mean squared error	Categorical cross-entropy
Batch size	8	8
Number of epochs	Maximum 30	Maximum 30
Early stopping	Validation loss, patience = 5, best weights restored	Validation loss, patience = 5, best weights restored
Evaluation metrics	MAE, Tolerance intervals	Accuracy, precision, recall, and F1-score

**Table 4:** Prediction performance and accuracy-based ranking of predictive models for raw sensory evaluation parameters.

Parameters	Rank	Model	Accuracy (Mean ± SD)		Precision	Recall	F1-Score
Water release	1	DenseNet121	0.902	±0.0360	0.878	0.821	0.834
	2	MobileNet	0.870	±0.0427	0.801	0.790	0.781
	3	InceptionV3	0.847	±0.0660	0.764	0.703	0.701
	4	VGG16	0.835	±0.0689	0.772	0.669	0.663
	5	VGG19	0.793	±0.0605	0.604	0.559	0.521
Texture	1	MobileNet	0.812	±0.0852	0.821	0.833	0.809
	2	DenseNet121	0.793	±0.1043	0.840	0.800	0.780
	3	InceptionV3	0.700	±0.0989	0.741	0.695	0.678
	4	VGG16	0.674	±0.1737	0.660	0.644	0.606
	5	VGG19	0.640	±0.1222	0.638	0.582	0.538
Odor	1	DenseNet121	0.756	±0.0632	0.753	0.709	0.692
	2	MobileNet	0.721	±0.0580	0.721	0.654	0.641
	3	InceptionV3	0.663	±0.1237	0.702	0.636	0.613
	4	VGG16	0.598	±0.1356	0.523	0.510	0.446
	5	VGG19	0.551	±0.0943	0.420	0.452	0.384
Color	1	DenseNet121	0.774	±0.0962	0.736	0.729	0.697
	2	MobileNet	0.728	±0.1140	0.765	0.704	0.670
	3	InceptionV3	0.691	±0.0760	0.690	0.644	0.607
	4	VGG16	0.584	±0.1810	0.382	0.427	0.352
	5	VGG19	0.563	±0.1526	0.295	0.367	0.289
Disintegration	1	DenseNet121	0.877	±0.0649	0.885	0.869	0.864
	2	MobileNet	0.856	±0.0942	0.864	0.850	0.834
	3	InceptionV3	0.853	±0.0581	0.854	0.840	0.836
	4	VGG16	0.784	±0.1320	0.786	0.758	0.737
	5	VGG19	0.747	±0.1193	0.748	0.694	0.670
Total Score	1	MobileNet	0.577	±0.1010	0.310	0.342	0.304
	2	DenseNet121	0.526	±0.1543	0.270	0.295	0.261
	3	InceptionV3	0.493	±0.1399	0.298	0.292	0.262
	4	VGG16	0.384	±0.1401	0.122	0.188	0.124
	5	VGG19	0.302	±0.0897	0.076	0.132	0.080
Purchasability	1	DenseNet121	0.926	±0.0500	0.914	0.899	0.891
	2	MobileNet	0.923	±0.0538	0.917	0.884	0.887
	3	VGG16	0.851	±0.0704	0.759	0.715	0.719
	4	InceptionV3	0.849	±0.0822	0.846	0.783	0.776
	5	VGG19	0.793	±0.0519	0.538	0.562	0.526

**Table 5:** Representative Grad-CAM visualizations for class-wise prediction of sensory quality parameters using the best-performing models.

Parameter	Actual Class	Original Image	Grad-Cam Heatmap	Grad-Cam Overlay	Predicted Class	Confidence
Water release	No water leakage	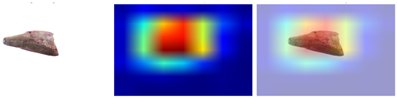			No water leakage	0.9996
	Water leakage present	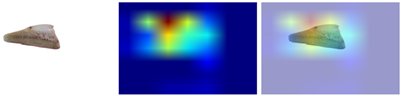			Water leakage present	0.5661
Texture	Firm	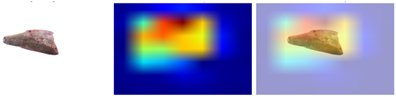			Firm	0.9998
	Slightly soft	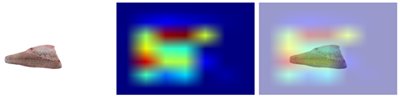			Slightly soft	0.8984
	Very soft	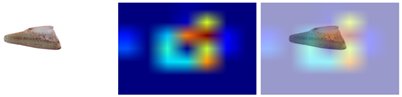			Very soft	0.5071
Odor	Fresh and neutral	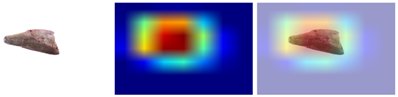			Fresh and neutral	0.9945
	Seaweed-like	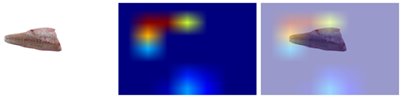			Sour-milk odor	0.4722
	Sour-milk odor	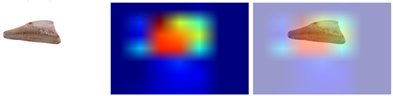			Sour-milk odor	0.5901
	Acetic acid and ammonia-like odor	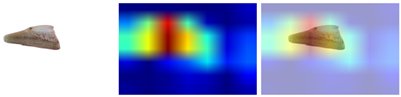			Acetic acid and ammonia-like odor	0.6861
Color	Characteristic color	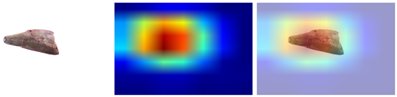			Characteristic color	0.9969
	Slightly yellow and light cream	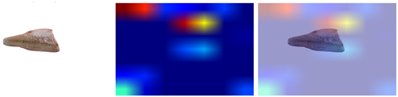			Slightly yellow and light cream	0.7354
	Dark yellow and cream	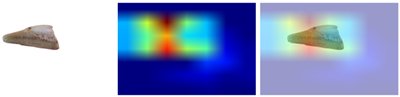			Dark yellow and cream	0.6070
Disintegration	Absent	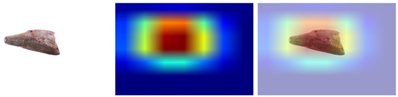			Absent	1.0000
	Present	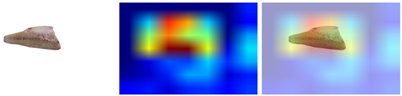			Present	0.9733
Total Score	0	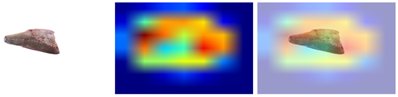			0	0.9476
	1	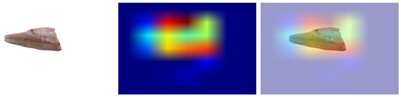			1	0.3595
	2	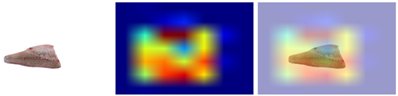			3	0.4181
	3	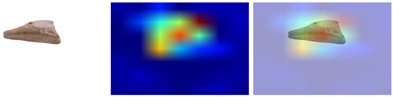			4	0.2415
	4	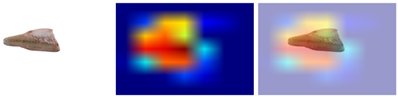			1	0.3355
	5	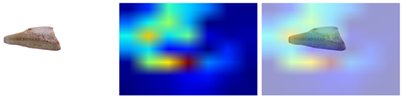			2	0.3874
	6	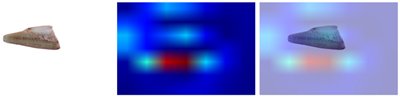			2	0.3458
	7	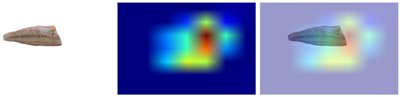			6	0.2647
	8	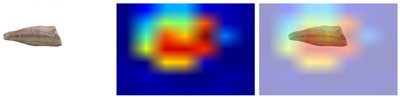			5	0.3970
	9	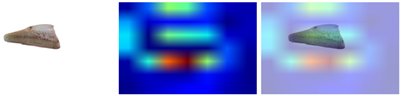			2	0.3216
Purchasability	Accept	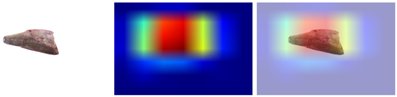			Accept	0.9983
	Reject	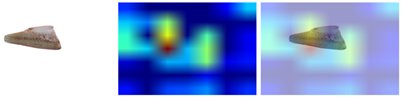			Reject	0.7213

**Table 6:** Prediction performance and MAE based ranking of predictive models for raw color analysis parameters (a*, b*, L*).

Parameters	Rank	Model	MAE (Mean ± SD)		Excellent	Good	Medium	Reject	Accept
Avg_a*	1	DenseNet121	0.680	±0.1154	8.37%	20.00%	22.33%	49.30%	50.70%
	2	MobileNet	0.718	±0.1574	8.84%	14.19%	25.35%	51.63%	48.37%
	3	VGG16	0.764	±0.2160	8.84%	16.74%	22.56%	51.86%	48.14%
	4	VGG19	0.797	±0.2261	7.91%	15.12%	22.56%	54.42%	45.58%
	5	InceptionV3	0.820	±0.2766	8.84%	14.42%	20.00%	56.74%	43.26%
Avg_b*	1	MobileNet	1.200	±0.1668	5.58%	8.37%	15.35%	70.70%	29.30%
	2	DenseNet121	1.206	±0.1812	4.19%	13.72%	11.86%	70.23%	29.77%
	3	InceptionV3	1.398	±0.2341	4.65%	8.37%	13.72%	73.26%	26.74%
	4	VGG16	1.510	±0.3922	3.95%	8.60%	11.86%	75.58%	24.42%
	5	VGG19	1.716	±0.3622	3.95%	7.21%	11.86%	76.98%	23.02%
Avg_L*	1	DenseNet121	2.467	±0.6675	2.56%	9.53%	13.95%	73.95%	26.05%
	2	MobileNet	2.593	±0.6376	1.63%	11.40%	11.63%	75.35%	24.65%
	3	InceptionV3	3.324	±0.7827	0.93%	8.37%	9.53%	81.16%	18.84%
	4	VGG16	4.015	±0.6661	0.47%	6.28%	6.51%	86.74%	13.26%
	5	VGG19	4.038	±0.5842	0.70%	7.67%	3.72%	87.91%	12.09%

**Table 7:** Prediction performance and MAE based ranking of predictive models for color analysis parameters (Chroma, Hue, Whiteness).

Parameters	Rank	Model	MAE (Mean ± SD)	
Avg_Chroma	1	MobileNet	1.024	±0.2278
	2	InceptionV3	1.053	±0.1302
	3	DenseNet121	1.054	±0.2910
	4	VGG16	1.160	±0.2775
	5	VGG19	1.250	±0.1982
Avg_Hue	1	DenseNet121	0.961	±0.1228
	2	VGG19	0.967	±0.1260
	3	VGG16	0.968	±0.1256
	4	MobileNet	0.995	±0.0928
	5	InceptionV3	1.003	±0.0709
Avg_Whiteness	1	DenseNet121	2.357	±0.5389
	2	MobileNet	2.585	±0.4704
	3	InceptionV3	3.221	±0.7348
	4	VGG16	3.926	±0.5878
	5	VGG19	4.006	±0.5899

**Table 8:** Robustness of the best-performing classification and regression models under simulated imaging variations.

	Parameter	Original	Low Brightness	High Brightness	Rotation	Condensation Blur	Glare Reflection
Accuracy	Water release	0.600	0.400	0.900	0.700	0.700	0.900
	Texture	0.867	0.867	0.867	0.600	0.400	0.800
	Odor	0.700	0.750	0.650	0.650	0.650	0.700
	Color	0.800	0.733	0.800	0.867	0.467	0.733
	Disintegration	0.800	0.800	0.800	0.800	0.800	0.800
	Total Score	0.280	0.220	0.280	0.160	0.100	0.220
	Purchasability	0.900	0.800	0.900	0.900	0.700	0.800
MAE	Avg_a*	0.620	0.663	0.588	0.551	0.564	0.559
	Avg_b*	0.975	1.024	0.881	1.038	0.707	1.409
	Avg_L*	1.860	2.224	1.457	1.466	10.907	1.389
	Avg_Chroma	0.622	0.530	0.698	0.863	0.576	0.720
	Avg_Hue	0.592	0.603	0.618	0.865	0.660	0.603
	Avg_Whiteness	2.352	2.683	1.907	1.731	10.819	1.441

## Data Availability

The dataset analyzed in this study was not retrieved from an existing repository but was generated specifically for this research using European seabass (*Dicentrarchus labrax*) samples purchased for experimental purposes. The dataset has not been deposited in a public repository; however, it may be obtained from the corresponding author upon reasonable request.

## Abbreviations

The following abbreviations are used in this manuscript:

a*	Red–green axis (in the color space)
AI	Artificial Intelligence
Avg	Average
b*	Yellow–blue axis (in the color space)
CIELab	International Commission on Illumination L*a*b* color space
CNNs	Convolutional neural networks
DHA	Docosahexaenoic acid
EPA	Eicosapentaenoic acid
FAO	Food and Agriculture Organization
FN	Number of positive test samples incorrectly predicted as negative
FP	Number of negative test samples incorrectly predicted as positive
K-value	A chemical freshness indicator based on ATP degradation products in fish
L*	Lightness axis (in the color space)
MAE	Mean absolute error
pH	Potential of hydrogen
TBARS	Thiobarbituric acid reactive substances
TMA	Trimethylamine
TN	Number of negative test samples correctly predicted as negative
TP	Number of test samples that are positive and are predicted as positive
TVB-N	Total Volatile Basic Nitrogen

## References

[1] FAO (2026). The State of World Fisheries and Aquaculture 2026—Blue Transformation: Turning Vision into Impact.

[2] Singh R.D., Arambam K., Singh A.M., Singh S.K. (2025). Health and nutritional benefits of fish as human food. Food Security, Nutrition and Sustainability Through Aquaculture Technologies.

[3] Hanna S., Greta K., Snježana Z., Jelka P. (2026). Nutritional Composition, Health Benefits and Claim Conditions of Fish from Aquaculture: A Narrative Review. Nutrients.

[4] Fuchiwaki Y., Yoshioka T. (2026). Evolution of freshness indicators and rapid sensing technologies for seafood: Toward a global platform for quality evaluation. Bull. Chem. Soc. Jpn..

[5] Rosati S., Maiuro L., Lombardi S.J., Iaffaldano N., Di Iorio M., Cariglia M., Lopez F., Cofelice M., Tremonte P., Sorrentino E. (2025). Integrated biotechnological strategies for the sustainability and quality of Mediterranean Sea bass (Dicentrarchus labrax) and Sea bream (Sparus aurata). Foods.

[6] Ali A., Wei S., Ali A., Khan I., Sun Q., Xia Q., Wang Z., Han Z., Liu Y., Liu S. (2022). Research progress on nutritional value, preservation and processing of fish—A review. Foods.

[7] Gleni K.E., Jesus B., Davies B.F.R., Pavlidis M., Barillé L. (2026). Evaluation of Post-Mortem Colour Changes in Fish Eye, Gills and Skin Using Hyperspectral Imaging. Aquac. Res..

[8] Das A., Dutta M.L., Sarma K.K. (2025). Fish Freshness Monitoring Technology: A Review. Sustainable Development, Innovation and Green Technology (ICASDIGT-2024).

[9] Basdeki E., Vasilaki S.E., Sensi M., Flemetakis E., Biscarini F., Power D., Tsironi T. (2025). Reviewing the correlation of fish quality alteration and in-package headspace composition: Evidence from a pH freshness indicator case study. Int. J. Food Sci..

[10] Chen Z., Li Y., Xie J., Mei J. (2026). Combined Analysis of Microbial Diversity, Water Migration, and Volatile Spoilage Profile Changes in Modified Atmosphere Packaged Crucian Carp (Carassius auratus) Fillets During Cold Storage. Food Biosci..

[11] Kundu M., Vashisth A., Krishnan P. (2026). Instrumental detection of fish freshness in research and food industry: A review. J. Food Sci. Technol..

[12] Huang G., Thongkaew K., Chaiprapat S. (2026). A Review of Non-Destructive Technologies for Quality Assessment in Aquaculture. Aquac. J..

[13] Singh R., Nickhil C., Nisha R., Upendar K., Jithender B., Deka S.C. (2025). A comprehensive review of advanced deep learning approaches for food freshness detection. Food Eng. Rev..

[14] Wang X., Feng Y., Wang Y., Zhu H., Song D., Shen C., Luo Y. (2025). Enhancing optical non-destructive methods for food quality and safety assessments with machine learning techniques: A survey. J. Agric. Food Res..

[15] Hamidy S.M., Kuvvetli Y., Sakarya Y., Özkütük S.T., Özoğul Y. (2025). Deep learning-based prediction of fish freshness and purchasability using multi-angle image data. Foods.

[16] Nazir A., Hussain A., Assad A. (2025). CNN in food industry: Current. Artificial Intelligence in the Food Industry: Enhancing Quality and Safety.

[17] Ding H., Hou H., Wang L., Cui X., Yu W., Wilson D.I. (2025). Application of convolutional neural networks and recurrent neural networks in food safety. Foods.

[18] Bonilla A.C., Sveinsdottir K., Martinsdottir E. (2007). Development of Quality Index Method (QIM) scheme for fresh cod (*Gadus morhua*) fillets and application in shelf life study. Food Control.

[19] Hunter R.S. (1975). Scales for the measurements of color difference. The Measurement of Appearance.

[20] Huang G., Liu Z., Van Der Maaten L., Weinberger K.Q. (2017). Densely connected convolutional networks. Proceedings of the IEEE Conference on Computer Vision and Pattern Recognition.

[21] Szegedy C., Vanhoucke V., Ioffe S., Shlens J., Wojna Z. (2016). Rethinking the inception architecture for computer vision. Proceedings of the IEEE Conference on Computer Vision and Pattern Recognition.

[22] Sakib S., Ahmed N., Kabir A.J., Ahmed H. (2018). An Overview of Convolutional Neural Network: Its Architecture and Applications. https://www.preprints.org/manuscript/201811.0546/v1.

[23] Zorgui S., Chaabene S., Bouaziz B., Batatia H., Chaari L. (2020). A convolutional neural network for lentigo diagnosis. International Conference on Smart Homes and Health Telematics.

[24] Howard A.G., Zhu M., Chen B., Kalenichenko D., Wang W., Weyand T., Andreetto M., Adam H. (2017). Mobilenets: Efficient convolutional neural networks for mobile vision applications. arXiv.

[25] Simonyan K., Zisserman A. (2014). Very deep convolutional networks for large-scale image recognition. arXiv.

[26] Hasanah S.A., Pravitasari A.A., Abdullah A.S., Yulita I.N., Asnawi M.H. (2023). A deep learning review of resnet architecture for lung disease Identification in CXR Image. Appl. Sci..

[27] Meena G., Mohbey K.K., Indian A., Kumar S. (2022). Sentiment Analysis from Images using VGG19 based Transfer Learning Approach. Procedia Comput. Sci..

[28] Grandini M., Bagli E., Visani G. (2020). Metrics for multi-class classification: An overview. arXiv.

[29] Şahin C., Balcı O., Işık M., Gökenç İ. (2023). Artificial neural networks approach for prediction of CIELab values for yarn after dyeing and finishing process. J. Text. Inst..

[30] Robeson S.M., Willmott C.J. (2023). Decomposition of the mean absolute error (MAE) into systematic and unsystematic components. PLoS ONE.

[31] Wang S., Das A.K., Pang J., Liang P. (2022). Real-time monitoring the color changes of large yellow croaker (Larimichthys crocea) fillets based on hyperspectral imaging empowered with artificial intelligence. Food Chem..

